# Perfect Detection of Spikes in the Linear Sub-threshold Dynamics of Point Neurons

**DOI:** 10.3389/fninf.2017.00075

**Published:** 2018-01-05

**Authors:** Jeyashree Krishnan, PierGianLuca Porta Mana, Moritz Helias, Markus Diesmann, Edoardo Di Napoli

**Affiliations:** ^1^Institute of Neuroscience and Medicine (INM-6) and Institute for Advanced Simulation (IAS-6) and JARA Institute Brain Structure Function Relationship (INM-10), Jülich Research Centre, Jülich, Germany; ^2^Aachen Institute for Advanced Study in Computational Engineering Science, RWTH Aachen University, Aachen, Germany; ^3^Institute for Advanced Simulation, Jülich Research Centre, Jülich, Germany; ^4^Department of Physics, Faculty 1, RWTH Aachen University, Aachen, Germany; ^5^Department of Psychiatry, Psychotherapy and Psychosomatics, Medical Faculty, RWTH Aachen University, Aachen, Germany

**Keywords:** state-space analysis, NEST, time-driven, event-driven, simulation, LIF neuron, differential geometry

## Abstract

Spiking neuronal networks are usually simulated with one of three main schemes: the classical time-driven and event-driven schemes, and the more recent hybrid scheme. All three schemes evolve the state of a neuron through a series of checkpoints: equally spaced in the first scheme and determined neuron-wise by spike events in the latter two. The time-driven and the hybrid scheme determine whether the membrane potential of a neuron crosses a threshold at the end of the time interval between consecutive checkpoints. Threshold crossing can, however, occur within the interval even if this test is negative. Spikes can therefore be missed. The present work offers an alternative geometric point of view on neuronal dynamics, and derives, implements, and benchmarks a method for perfect retrospective spike detection. This method can be applied to neuron models with affine or linear subthreshold dynamics. The idea behind the method is to propagate the threshold with a time-inverted dynamics, testing whether the threshold crosses the neuron state to be evolved, rather than vice versa. Algebraically this translates into a set of inequalities necessary and sufficient for threshold crossing. This test is slower than the imperfect one, but can be optimized in several ways. Comparison confirms earlier results that the imperfect tests rarely miss spikes (less than a fraction 1/10^8^ of missed spikes) in biologically relevant settings.

## 1. Introduction

In the last decade considerable work has been devoted to improving the accuracy of efficient simulators of large networks of spiking neurons (Hansel et al., 1998; Mattia and Del Giudice, 2000; Shelley and Tao, 2001; Dehaene and Changeux, 2005; Brette, 2007; Morrison et al., 2007; D'Haene et al., 2009; van Elburg and van Ooyen, 2009; Zheng et al., 2009; Hanuschkin et al., 2010). This research field is driven by the ideal of combining two antagonistic goals: coping with a realistically high frequency of synaptic events arriving at a neuron, as in nature, and at the same time implementing in a mathematically accurate way the threshold process on which a wide class of neuron models is based.

Two classical schemes to simulate neuronal networks are the time-driven and the event-driven schemes (Ferscha, 1996; Fujimoto, 2000; Zeigler et al., 2000). Both schemes describe the state of the neurons by a set of variables and the action potentials as events that mediate the interaction between them.

In a time-driven scheme, the state of a neuron is updated on a time grid defined by the simulation step (for a review see Morrison and Diesmann, 2008). After all neurons are updated, their membrane potential is checked for threshold crossings. If the membrane potential of a neuron is above the threshold at this checkpoint, a spike is delivered to all neurons it is connected to, and the potential is reset. Then a new iteration step begins. The step size stipulates how frequently the threshold-crossing inspections occur during the simulation. The choice of the step size trades off the spike-detection accuracy against the simulation speed (Morrison et al., 2007). Such grid-constrained simulations force each spike event to a position on the equispaced temporal grid spanned by the step size and therefore induce artificial synchronization of the network dynamics (Hansel et al., 1998; Shelley and Tao, 2001; Brette, 2007; Morrison et al., 2007; van Elburg and van Ooyen, 2009; Hanuschkin et al., 2010).

Event-driven schemes correct such artificial synchronization. In an event-driven scheme the state of a neuron is updated only when it receives a spike. A central queue of events is maintained and each spike is inserted into this queue with its own time stamp. Upon update a neuron predicts when its next spike will occur in the absence of further input. This preliminary event is inserted into the queue and confirmed if it becomes due or removed when invalidated by further input. Efficient and elegant spike prediction methods have been developed for classes of non-invertible neuron dynamics (Ferscha, 1996; Brette, 2007; D'Haene et al., 2009; van Elburg and van Ooyen, 2009; D'Haene and Schrauwen, 2010). Maintaining a central queue in a distributed simulation is challenging, however, and may compromise the time performance of the simulator (for a detailed review see Hanuschkin et al., 2010).

A hybrid scheme circumvents the shortcomings of time-driven and event-driven schemes by embedding a locally event-driven algorithm for each neuron into a globally time-driven scheme (Morrison et al., 2007). The arrival of a spike at a neuron introduces an additional update and checkpoint besides the global checkpoints. The motion of a given neuron in state space is then propagated from incoming spike to incoming spike and eventually to the end point of the global timestep. If the membrane potential of a neuron is above the threshold at a local or global checkpoint, the precise point of threshold crossing is determined in continuous time, and a spike is emitted. In this scheme spike events carry a floating point offset next to their location on the time grid. Thus, in contrast to an event-driven scheme, the hybrid scheme does not predict future spike times but identifies threshold crossings only retrospectively. Hanuschkin et al. (2010) demonstrate that the retrospective scheme is as accurate as the predictive one but at lower computational costs.

The hybrid scheme still has a loophole, however: spikes can be missed. The reason is that the crossing of the threshold voltage is tested by inspecting whether the membrane potential *V* of the neuron is above threshold θ at the end of a checkpoint, *t*_0_ + *h*, as in the time-driven scheme:

(1) $$\begin{array}{l} V\left(t_{0}+h\right)⩾\theta \Rightarrow \text{spike}. \end{array}$$

**Figure 1:**
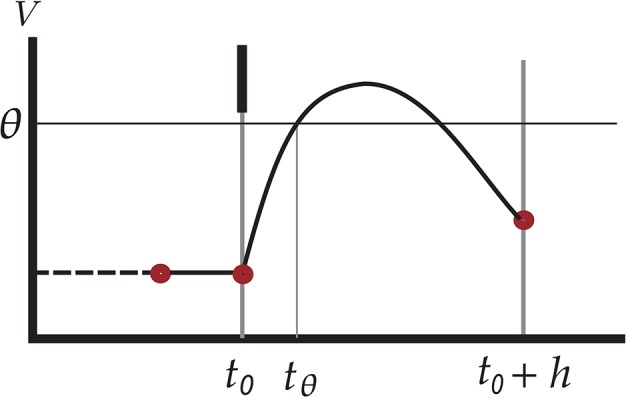
Illustration of undetected threshold crossing between two consecutive checkpoints *t*_0_ and *t*_0_ + *h* on the time grid. The short black vertical bar represents an incoming spike which causes an increase in the membrane voltage of the neuron, leading to a threshold crossing at *t*_θ_. The subthreshold dynamics, however, brings the voltage under threshold again at the next checkpoint. Since the test *V*(*t*_0_ + *h*) ⩾ θ yields false, the outgoing spike at *t*_θ_ is missed. The red dots indicate points where the values of state variables are known.

See Figure 1 for an illustration of this scenario. However, the evolved membrane potential can be below threshold at the end of a checkpoint and yet have crossed the threshold two, four, 2*n* times between the initial and end checkpoints *t*_0_ and *t*_0_ + *h*. The first crossing constitutes a missed spike. Symbolically,

(2) $$\begin{array}{l} V\left(t_{0}+h\right)⩾\theta \Rightarrow \text{threshold crossing},\text{ but }\\ \text{threshold crossing}⇏V\left(t_{0}+h\right)⩾\theta \end{array}$$

so the test *V*(*t*_0_ + *h*) ⩾ θ is a sufficient but not necessary condition for the occurrence of a threshold crossing during the time interval ]*t*_0_, *t*_0_ + *h*]. For future reference we call this test *standard test*.

The question remains whether a globally time-driven scheme can formulated such that it detects every threshold crossing. Although Hanuschkin et al. (2010) argue that the loss of spikes of the standard test is not of practical relevance in natural parameter regimes, the availability of an absolutely lossless method would free the researcher from inquietude and costly controls when faced with previously unexplored neuron models or network architectures.

In this work, we propose a new spike-detection method based on a necessary and sufficient condition for threshold crossing to occur in a given time interval; we therefore call it *lossless method* or *lossless test*. The method is derived from state-space analysis and works especially well with neuron models with affine or linear subthreshold dynamics; i.e., of the form $\dot{s}\left(t\right)=As\left(t\right)+q$ or $\dot{s}\left(t\right)=As\left(t\right)$. It consists of a system of inequalities—some linear, some non-linear in the state-space variables—that together determine whether the initial state of a neuron will or will not reach threshold within the time interval until the next checkpoint. This lossless method is meant to replace the standard test (Equation 1) in the time-driven and the hybrid scheme. Alone it does not solve the problem of artificial synchronization characteristic of time-driven schemes. Hence this method is most meaningful within a hybrid scheme. Thanks to its perfect spike detection the lossless method can in fact be used to benchmark hybrid schemes based on the standard test; Hanuschkin et al. (2010) use the method of D'Haene et al. (2009) for this purpose.

In section 3 we present the idea behind the lossless method for an affine or linear neuron dynamics, and develop its mathematical construction. Parts of this construction must be addressed on a case-by-case basis; therefore in section 4 we provide a concrete implementation of the lossless method for the leaky integrate-and-fire model with exponential synaptic currents (Fourcaud and Brunel, 2002), within the hybrid scheme. Readers interested only in the implementation are encouraged to proceed directly to section 4.

The method can be algorithmically expressed in different ways. We explore two alternative sequences of inequalities and assess their costs in terms of time-to-completion relative to each other and to the hybrid scheme based on the standard test. For the latter scheme we also assess the number of missed spikes in commonly considered network regimes. The hybrid scheme based on our lossless method delivers the desired exact implementation of the mathematical definition of the neuron model without any further approximation up to floating point precision.

Preliminary results have been published in abstract form (Kunkel et al., 2011; Krishnan et al., 2016). The technology described in the present article will be made available with one of next major releases of the open-source simulation software NEST. The conceptual and algorithmic work described here is a module in the long-term collaborative project to provide the technology for neural systems simulations (Gewaltig and Diesmann, 2007).

## 2. Idea: moving a surface backwards instead of a point forward

Let us summarize the problem mentioned in the previous section. We assume that a neuron's state evolves according to three different dynamical laws or motions in state-space: (a) an integrable subthreshold dynamical law as long as the neuron's membrane potential is below threshold and there are no changes in input currents; (b) discrete jumps in the subthreshold motion at predetermined times, corresponding to incoming spikes or to sudden changes in external currents; these can be formally incorporated into the subthreshold dynamical law via delta functions; (c) a “spike,” i.e., an instantaneous jump of the membrane potential from threshold to a reset value, as soon as the potential reaches the threshold value. The jump may be followed by a refractory period in which the membrane potential remains constant at the reset value. Then the integrable motion (a) takes place again.

The advantage of an integrable subthreshold dynamical law is that the state of the neuron at a time *t*_0_ + *h* can be analytically determined by that at time *t*_0_; here *h* can be negative or positive, and for simplicity we set *t*_0_ = 0 in what follows. The evolution can thus be calculated in discrete time steps, in particular in between times at which jumps (b) occur. The spiking component of the motion, however, forces us to check whether the membrane potential *V* reached a threshold value θ within the timestep interval ]0, *h*]. We call this event *threshold crossing*; by “crossing” we also mean tangency. A sufficient condition for threshold crossing is that the membrane potential be above threshold at the end *h* of the time step: by continuity, it must have assumed the threshold value at some time in the interval ]0, *h*]. But, as mentioned in the introduction, this condition is not necessary: during the time step the potential may touch or surpass the threshold value and then go below it again, as in Figure 1, an even number of times. Its value is then below threshold at both ends of the time step, *t* = 0 and *t* = *h*. A test that only relies on the sufficient condition *V*(*h*) ⩾ θ, like the standard test, can therefore miss some spikes, leading to an incorrect motion in state-space. We need a test based on a necessary and sufficient condition.

**Figure 2:**
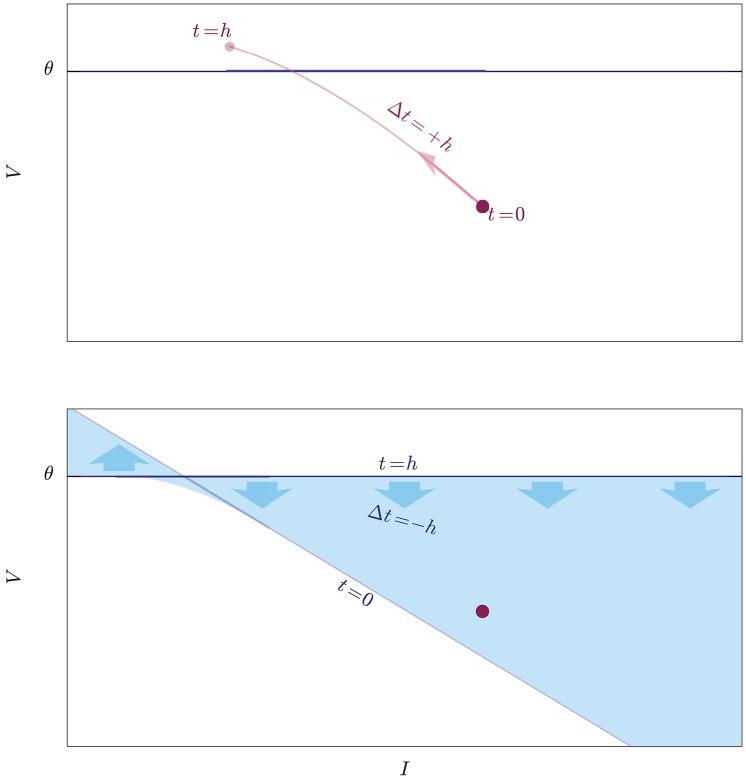
Illustration of two exact methods to check whether an initial state (red dot) crosses the threshold (blue horizontal line) during the evolution from *t* = 0 to *t* = *h*. **Upper:** The idea of a root-finding method is to evolve the state forwards in time by a step Δ*t* = +*h*, and to check whether its trajectory (light-red curve) intersects the threshold. **Lower:** The idea of the lossless method is to evolve the whole threshold “backwards in time” from *t* = *h* to *t* = 0 by a step Δ*t* = −*h* and to check whether its trajectory, which is a volume in state-space (light-blue region), contains the initial state, which is kept fixed. The threshold shifts and rotates as it evolves, and the trajectories of its individual points are unknown: this method does not inform us of when and where on the crossing it occurs, and is therefore computationally faster.

The threshold is a hyperplane in state space with equation *V* = θ, and “threshold crossing” means that the trajectory of the state during ]0, *h*], a curve in state space, intersects this hyperplane. This is equivalent to looking for the roots of the equation *V*(*t*)−θ = 0, checking whether this set of roots is empty or complex (no threshold crossing) or non-empty and real (threshold crossing). This idea is illustrated in Figure 2 for a two-dimensional state space. Such equation is usually transcendental and its roots have to be found numerically (Press et al., 2007, ch. 9). As one of the first steps in this root search, “you should always bracket a root, that is, know that the function changes sign in an identified interval, before trying to converge to the root's value” (Press et al., 2007, § 9.0); and this is what the necessary but insufficient condition *V*(*h*) ⩾ θ does. When this condition is not met, a necessary and sufficient condition is that the maximum of the voltage *V*(*t*) be above threshold for *t* ∈ ]0, *h*]. This would leads us to maximization algorithms (Press et al., 2007, ch. 10).

We want to approach this problem from a point of view that mathematically is equivalent to checking whether the maximum of the voltage *V*(*t*) in the time interval *t* ∈ ]0, *h*] is above threshold, but geometrically is very different. Up to now we have imagined to move the initial state of the neuron at time *t* = 0, which is a point in state space, and to check whether this moving point touches the threshold hyperplane before time *t* = *h*. All the while the threshold hyperplane has been at rest, so to speak. This is like filming a runner rushing toward the finish line, by means of a video camera stationed beside the latter. But the same problem can be looked at from a different frame of reference: we can keep the state of the neuron at rest, and move the whole threshold (i.e., the states that lie on the threshold) instead, for the same amount of time, and check whether this moving hypersurface touches the point. This is like having a video camera fastened on the runner, filming the approaching finish line. As long as the *relative* motion of the two objects is the same, the question of whether they meet has the same answer whether we see the one moving while the other is at rest, or vice versa. The two frames of reference are illustrated in Figure 2 for a two-dimensional state space.

In the second reference frame, the propagation of the points of the threshold has a dynamical law where time appears with a negative sign (see section 3.2), as if these points were moving backwards in time. For this reason, as a visual aid, we picturesquely call the first point of view “propagation of the state forwards in time,” and the second “propagation of the threshold backwards in time,” or just “backpropagation.”

In the first frame of reference we are checking whether a curve (the trajectory of the state) intersects a hypersurface (the threshold). In the second frame of reference we are checking whether a hypervolume (the trajectory of the threshold) intersects a point (the state); in other words we are checking whether a point belongs to a particular state-space region. The test for the intersection of a 1-dimensional curve with an (*N*−1)-dimensional surface is replaced by the test for the membership of a point in an *N*-dimensional volume.

Geometrically the latter test translates into a system of *inequalities* that the initial state at *t* = 0 must satisfy if it does not cross threshold within the time interval ]0, *h*]. This means that the maximum of the voltage *V*(*t*) in that same interval is below threshold.

The equations corresponding to these inequalities represent the boundary between the set of states that will cross the threshold within the timestep *h*—which we call *spike region*—and the set of those that won't—which we call *no-spike region*. Finding these inequalities in explicit form is the most important point of this method, and can be achieved with this heuristic procedure:

- Find, in parametric form, the hypervolume swept by the moving threshold. This is done by representing the propagation of the threshold in state-space as a map between two manifolds: the product manifold threshold × time, and the state-space manifold.
- Find the boundary of this hypervolume, in parametric form. This is done by determining the placement of the images of the boundaries of the product manifold and, most important, of the images of the critical points of the map. The latter are the points at which the map becomes singular, thus mapping *N*-dimensional regions to (*N*−1)-dimensional ones (Choquet-Bruhat et al. 1996, § II.A.1; Spivak 1999, ch. 2).
- Transform the equations of the boundary above from parametric to implicit form. If this is not possible, the boundary can still be approximated by a simplicial mesh, as finely as we please.
  At this point we have a set of inequalities that can separate the states that will spike from those that won't.
- Finally, optimize the resulting set of inequalities, by e.g., adding to them a preliminary set of linear inequalities that delimit the larger parts of the spike or no-spike regions.

In the following analysis we mathematically develop the first two steps in a general way for any affine neuron dynamics. They can be generalized to other kinds of dynamics; we get back to this point in section 5. The procedure for the last two steps depends on the particular neuron model, so we can only give general guidelines. section 4 provides a concrete example of all steps. An advantage of our algorithm is that it its construction needs to be carried out only once for any given neuron model.

Our algorithm tells whether a neuron state will cross the threshold within a time increment *h*. By construction it does not tell at what time *t*_θ_ the crossing, if any, occurs. To determine such time we must call a root-finding algorithm (Press et al., 2007, ch. 9); but our algorithm has already bracketed the root to be found.

We believe that our geometric, state-space point of view can more readily suggest possible optimizations for the threshold-crossing test than a point of view focused on state trajectories and voltage maxima, as we will show in section 4.

## 3. A time-reversed state-space analysis

### 3.1. Mathematical preliminaries

The final equations to be obtained, Equation (25), can be derived by concepts from vector analysis, Cartesian geometry, and functional analysis; but the derivation is lengthy. To shorten it we use concepts and terminology from affine spaces (Artin, 1955; Coxeter, 1969; Rockafellar, 1972; Nomizu and Sasaki, 1994; Porta Mana, 2011), and differential manifolds (de Rham, 1984; Burke, 1987; Schouten, 1989; Bossavit, 1991, 2002; Dodson and Poston, 1991; Simon et al., 1992; Nomizu and Sasaki, 1994; Burke, 1995; Choquet-Bruhat et al., 1996; Ramanan, 2005; Marsden and Ratiu, 2007).

The state space *S* of a neuron has a natural vector-space structure (an affine-space structure would also suffice), inherited from the physical quantities that define it: the membrane potential *V* and other *N* − 1 physical quantities ***I*** whose exact number and definition depend on the specific neuron model (e.g., ***I*** could represent currents or additional voltages, for example of different compartments).

Every hyperplane in the state-space is defined by an affine equation ***k***^**⊺**^***s*** = κ, the covector ***k***^**⊺**^ being the normal to the hyperplane, where κ is the affine term. The inequality ***k***^**⊺**^***s*** > −κ defines one of the two half-spaces delimited by the hyperplane. The threshold hyperplane is especially important: it is the set of states ***s*** whose membrane potential has the threshold value: *N*-dimensional differential manifold: its points {***s***} are the neuron states, and the quantities (***I***, *V*) are coordinates *V* : *S* → **R** and ***I*** : *S* → **R**^*N*−1^ e.g., the membrane potential of a state ***s*** is *V*(***s***). These coordinates respect the vector structure of the state-space, i.e., *V*(***s***_1_ + ***s***_2_) = *V*(***s***_1_) + *V*(***s***_2_) and likewise for ***I***.

(3) $$\begin{array}{l} V\left(s\right)=\theta . \end{array}$$

Its equation ***k***^**⊺**^***s*** = κ in coordinates (***I***, *V*) has coefficients

(4) $$\begin{array}{l} k^{⊺}=\left(0^{⊺},1\right),\;\kappa =\theta ,\\ k^{⊺}s=\kappa \text{ on threshold},\;k^{⊺}s<\kappa \text{ below threshold}. \end{array}$$

An affine transformation of the state-space onto itself,

(5) $$\begin{array}{l} s\mapsto Ms+m, \end{array}$$

where ***M*** is a linear transformation and ***m*** a state, maps each hypersurface and half-space ***k***^**⊺**^***s*** ⩾ κ to a hypersurface and half-space ***k***′^**⊺**^***s*** ⩾ κ′ with

(6) $$\begin{array}{l} {k^{\prime }}^{⊺}=k^{⊺}M^{-1},\;\kappa^{\prime }=\kappa +k^{⊺}M^{-1}m \end{array}$$

(the transformation of the normal ***k***^**⊺**^ shows why it is a covector rather than a vector).

We now show that the integrable dynamical law within a finite time step *h* generates affine transformations. Consider the evolution equation

(7) $$\begin{array}{l} \dot{s}\left(t\right)=As\left(t\right)+q. \end{array}$$

In a time interval *h*, an initial state ***s***_0_ propagates under this evolution into the final state at time *t*_0_

(8) $$\begin{array}{l} s\left(t_{0}+h\right)={\text{e}}^{hA}s\left(t_{0}\right)+\left({\text{e}}^{hA}-1\right)A^{-1}q,\;s\left(t_{0}\right)=s_{0}. \end{array}$$

This, for each *h*, is an affine transformation of the form (4). In coordinates (***I***, *V*) the linear operator ***A*** and vector ***q*** have the block form

(9) $$A=\left(\begin{array}{cc} B & d\\ c^{⊺} & \alpha \end{array}\right),\;q=\left(\begin{array}{c} r\\ \beta \end{array}\right),$$

where ***B*** is an (*N* − 1, *N* − 1) matrix, α and β numbers, and the dimensionalities of the rectangular matrices ***c***^**⊺**^, ***d*** follow accordingly.

### 3.2. Derivation of the threshold-crossing condition

Let us mathematically summarize the first threshold-crossing test discussed in section 2. We said that the state evolution (Equation 8) can be efficiently used in a time-step scheme in numerical simulations, but we need to test whether a threshold crossing occurred at some time *t* ∈ ]*t*_0_, *t*_0_ + *h*]. A necessary and sufficient condition would be the existence of solutions of the transcendental equation in *t*

(10) $$\begin{array}{l} V\left(e^{tA}s\left(t_{0}\right)+\left(e^{tA}-1\right)A^{-1}q\right)=\theta ,\;t\in ]0,h], \end{array}$$

which corresponds to the intersection of the trajectory (Equation 8) and the threshold hyperplane (Equation 3); but it is a costly condition to test.

We now develop the second kind of threshold-crossing test discussed in section 2, according to the steps I–IV. Steps I and II are performed for any affine dynamics. Steps III and IV have to be solved on a case-by-case basis, so their analysis below is only a guideline; a concrete example on how to perform them is given in section 4.2.3 and 4.2.4.

#### 3.2.1. Hypervolume in parametric form

Consider the threshold hyperplane *V*(***s***) = θ as an (*N* − 1)-dimensional manifold with coordinates ***x***. It is embedded in state-space via the map

(11) $$\begin{array}{l} x\mapsto \left(I,V\right)=\left(x,\theta \right). \end{array}$$

**Figure 3:**
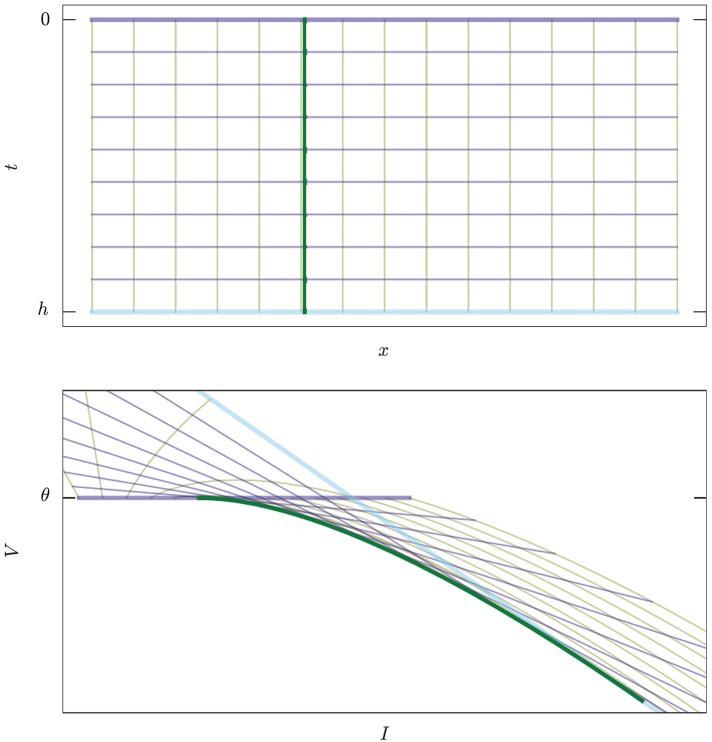
Example of map *E* : (***x***, *t*) ↦ (***I***, *V*), Equation (12), in two dimensions. **Upper:** The abstract manifold with coordinates (***x***, *t*) corresponding to the values of current and time to threshold crossing. **Lower:** The image of the map in state space. Thick green curve corresponds to the set of singular points where det(*TE*) = 0, Equation (10). Yellow lines, constant ***x***, are trajectories of states ending on the threshold. Violet lines, constant *t*, are snapshots of the threshold moving “backwards in time.” The thicker violet and blue lines correspond to the boundaries *t* = 0 and *t* = *h*. For *t* = 0 we have *I* = *x*, *V* = θ.

Each state (***x***, θ) on the threshold, when propagated backwards in time for an interval *h*, traces a curve in state-space (the yellow lines of Figure 3).

**Figure 4:**
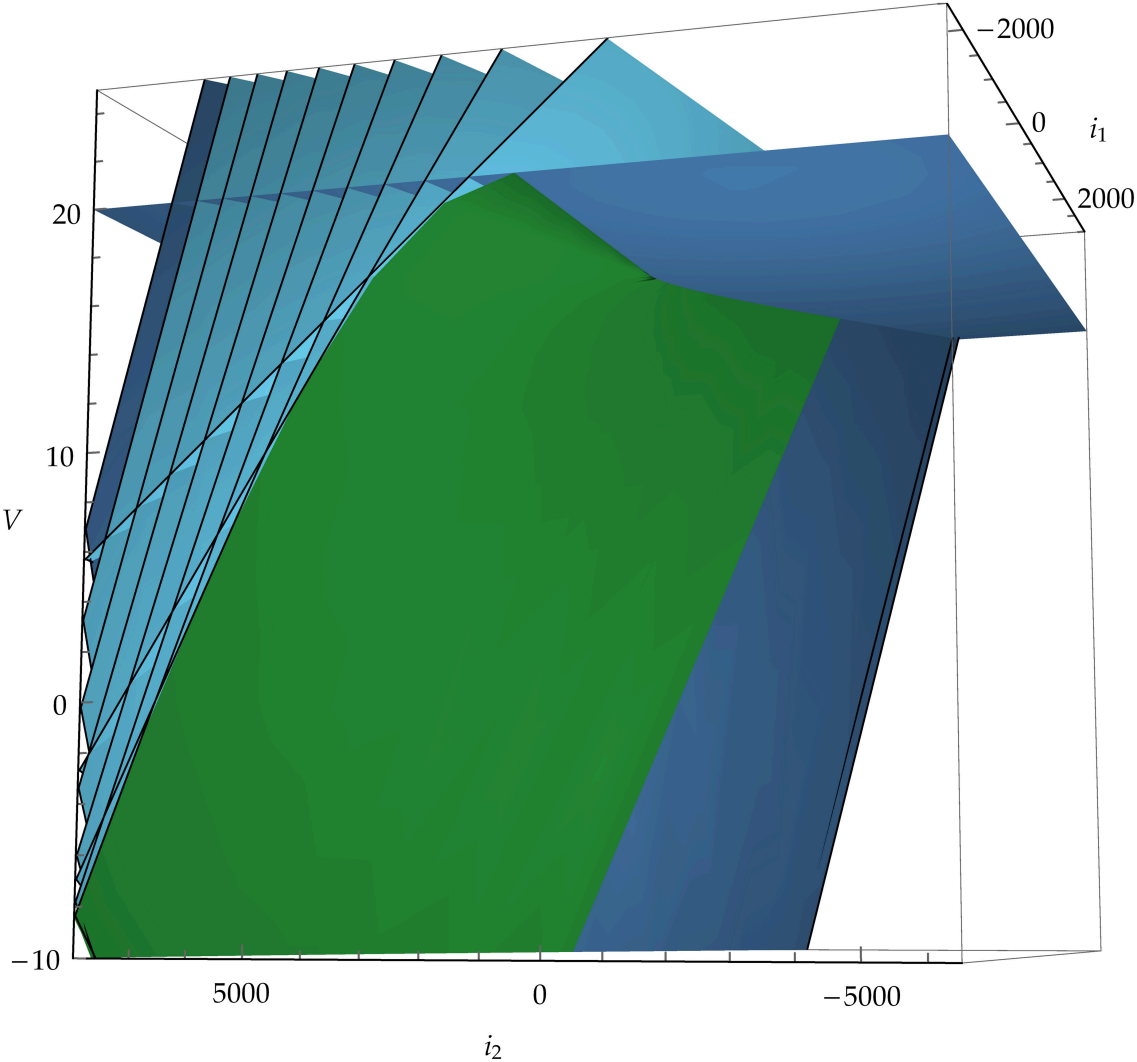
Example of the image of the map *E* : (***x***, *t*) ↦ (***I***, *V*), Equation (12), in a three-dimensional state space. Blue planes are snapshots of the threshold plane (horizontal, darker blue plane) moving “backwards in time.” The green, curved surface corresponds to the set of singular points where det *E*′ = 0, Equation (10). The neuron model in this example is leaky integrate-and-fire with α-shaped post-synaptic currents *i*_1_, *i*_2_ and membrane potential *V*.

The union of these curves is an *N*-dimensional product manifold, called the *extrusion* (cf. Chap. 5. Bossavit, 2003) of the threshold hyperplane. We can use coordinates (***x***, *t*) ∈ (**R**^*N* − 1^ × [0, *h*]) on this manifold. Its mapping into state-space is given, with the help of Equation (8), by

(12) $$\begin{array}{l} E:\left(x,t\right)\mapsto {\text{e}}^{-tA}\left(\begin{array}{c} x\\ \theta \end{array}\right)+\left({\text{e}}^{-tA}-1\right)A^{-1}\left(\begin{array}{c} r\\ \beta \end{array}\right), \end{array}$$

where *t* > 0 is the direction of the past. This map is analytic, but generally not an *embedding* because it can have self-intersections. We will see the significance of this in section 3.3. It is not an *immersion* either because it can have singular points; these will be especially important for us because they constitute part of the boundary between spike and no-spike regions. See Figure 3 for a two-dimensional example, and Figure 4 for a three-dimensional one.

For fixed *t*, the map ***x*** ↦ *E*(***x***, *t*) is affine, and its image is a hyperplane representing the states on the set of states at threshold propagated backwards in time for an interval *t*. Combining it with Equations (4–6) we find that the set of states of backpropagated threshold state has *t*-dependent normal and affine terms

(13) $$\begin{array}{l} \begin{array}{c} k_{t}^{⊺}=\left(\begin{array}{c} 0^{⊺},1 \end{array}\right){\text{e}}^{tA}\\ \kappa_{t}=\theta +\left(\begin{array}{c} 0^{⊺},1 \end{array}\right)\left(1-{\text{e}}^{tA}\right)A^{-1}\left(\begin{array}{c} r\\ \beta \end{array}\right). \end{array} \end{array}$$

The inequality $k_{t}^{⊺}s<\kappa_{t}$ determines the backpropagated half-space, which is below threshold.

#### 3.2.2. Hypervolume boundary in parametric form

We must now find the boundary of the image of the map *E*. The latter is a closed set, being the image of a closed set under a continuous map; its boundary must therefore be the image of some points of the domain. Such points must either lie on the boundaries of the domain, **R**^*N* − 1^ × {0} and **R**^*N* − 1^ × {*h*}, or be critical points of *E*, or both, because *E* is differentiable. See the example of Figure 3.

The images of the boundary are easily found from (Equation 12): one (image of *t* = 0) is the threshold hyperplane, the other (*t* = *h*) is the hyperplane $k_{h}^{⊺}s=\kappa_{h}$, with coefficients given by Equation(33). Explicitly, in coordinates (***I***, *V*),

(14) $$\begin{array}{l} V=\theta , \end{array}$$

(15) $$\begin{array}{l} \left(\begin{array}{c} 0^{⊺},1 \end{array}\right){\text{e}}^{hA}\left(\begin{array}{c} I\\ V \end{array}\right)=\theta +\left(\begin{array}{c} 0^{⊺},1 \end{array}\right)\left(1-{\text{e}}^{hA}\right)A^{-1}\left(\begin{array}{c} r\\ \beta \end{array}\right). \end{array}$$

Let us find the image of the critical points of *E*. The derivative of *E* at a point (***x***, *t*) is

(16) $$\begin{array}{l} E^{\prime }\left(x,t\right)=-{\text{e}}^{-tA}\left(\begin{array}{cc} I & Bx+d\theta +r\\ 0^{⊺} & c^{⊺}x+\alpha \theta +\beta \end{array}\right). \end{array}$$

This is also called the tangent map of *E*, and denoted E_*_, D*E*, or T*E* in differential-geometry texts.

Its determinant, the Jacobian determinant of the map, represents the inverse ratio between a volume element at that point and its image in state-space (the sign determines their relative orientation). Hence this ratio vanishes at points where volume elements are mapped onto area elements (in other words, *N* linearly independent vectors in the domain are mapped onto *N* linearly dependent vectors), which is a feature of the boundary. See the two-dimensional example of Figure 3.

Let us look for points where det*E*′(***x***, *t*) = 0. In Equation (16), the determinant of the exponential never vanishes, so we only have to consider the determinant of the matrix on the right. This is easily calculated by Laplace expansion along the last row, whose elements all vanish except the last. The cofactor of the last element is det ***I*** (modulo a sign). Hence

(17) $$\begin{array}{l} \det E^{\prime }\left(x,t\right)=0\;\Leftrightarrow \;c^{⊺}x+\alpha \theta +\beta =0 \end{array}$$

and the coordinates (***x***, *t*) of critical points satisfy

(18) $$\begin{array}{l} c^{⊺}x+\alpha \theta +\beta =0,\;0⩽t⩽h. \end{array}$$

This equation says that one of the coordinates ***x*** has an affine dependence on the remaining ones; let us call these ***y***. For example, if *c*_*N*−1_ ≠ 0, the equation above has the parametric solution

(19) $$\begin{array}{l} \begin{array}{c} x_{1}=y_{1},\;\ldots ,\;x_{N-2}=y_{N-2},\\ x_{N-1}=-\frac{c_{1}y_{1}+\cdots +c_{N-2}y_{N-2}+\beta +\alpha \theta }{c_{N-1}}. \end{array} \end{array}$$

Denote this affine dependence by ***x***(***y***). By taking the derivative of Equation (18) with respect to ***y*** we have

(20) $$\begin{array}{l} c^{⊺}\partial_{y}x=0^{⊺}, \end{array}$$

a property we will use later.

The locus of critical points in state-space is then given parametrically by a map ***Γ***, found by substitution of Equations (19) in Equation (12):

(21) $$\begin{array}{l} \Gamma :\left(y,t\right)\mapsto {\text{e}}^{-tA}\left(\begin{array}{c} x\left(y\right)\\ \theta \end{array}\right)+\left({\text{e}}^{-tA}-1\right)A^{-1}\left(\begin{array}{c} r\\ \beta \end{array}\right),\;0<t<h. \end{array}$$

This locus has four important features:

1. The points on the locus corresponding to fixed *t* belong to a hyperplane with coefficients (Equation 33), as is easily checked by substitution. In other words, the locus of critical points is the envelope of the backpropagated threshold hyperplanes $k_{t}^{⊺}s=\kappa_{t}$, Equation (33), at different times *t*, and its tangent hyperplanes have normals $k_{t}^{⊺}$ given by Equation (33). In particular, this locus is tangent to the threshold hyperplane.
2. Comparing the dynamical law (Equation 7), the map for the threshold hyperplane (Equation 11), and the critical-point condition (Equation 18), we notice that the latter is also the condition for the trajectory (Equation 8) of a state ***s***_0_ = (***x***, θ) on the threshold hyperplane to have an extremum in the membrane potential *V*. Hence, *the locus of critical points is the trajectory of the intersection between the threshold and the V-nullcline*.
3. From the form of Equations (18, 21), the locus of critical points is flat along *N*−2 dimensions—corresponding to a fixed value of the coordinate *t*—and is curved normally to the direction *t*: *it is an* (*N*−2)*-ruled surface*.
4. The dynamical law (Equation 7) preserves affine combinations of solutions—and therefore convex combinations as well. If ***s***_1_, ***s***_2_ are two arbitrary initial states in the no-spike region, then their propagated states also satisfy *V*[***s***_1_(*t*)] < θ and *V*[***s***_2_(*t*)] < θ when 0 ⩽ *t* ⩽ *h*; and also the propagation of their convex combination λ***s***_1_ + (1 − λ)***s***_2_, 0 < λ < 1 satisfies
  (22) $$\begin{array}{l} V\left(\lambda s_{1}\left(t\right)+\left(1-\lambda \right)s_{2}\left(t\right)\right)<\theta ,\;0<\lambda <1, \end{array}$$
  i.e., it lies in the no-spike region. This proves that the no-spike region is *convex*. This also means that the locus of critical points is a *concave*, i.e., ∩-shaped, surface: the segment between any two of its points lies completely within the no-spike region.

In view of the first property above, let us call the locus of critical points *envelope*, for brevity.

#### 3.2.3. Hypervolume boundary in implicit form or its approximation

The next step is the elimination of the parameters (***y***, *t*) to express the envelope ***Γ*** as one or more implicit equations χ(***s***) = 0 defined on domains *D*_χ_ for the state-space coordinates ***s***. This step can involve a transcendental equation, and a closed-form solution for it may not exist. We must therefore proceed in two possible ways depending on its existence:

- If an implicit, closed form for the envelope ***Γ*** exists, we can find a function χ of the state-space variables such that χ(***s***) = 0 is the envelope:
  (23) $$\begin{array}{l} \left\{s|\chi \left(s\right)=0,s\in D_{\chi }\right\}\;=\;\left\{\Gamma \left(y,t\right)|y\in {\text{R}}^{N-2},0⩽t⩽h\right\}. \end{array}$$
  Since χ is determined apart from a multiplicative constant, we can choose this constant in such a way that the inequality χ(***s***) < 0, ***s*** ∈ *D*_χ_, determines the region below the hypersurface contained in the no-spike region.
  It is sometimes possible to find an implicit closed-form for the envelope ***Γ*** even when a closed-form solution for the intersection of a propagated state and the threshold does not exist. The concrete model discussed in section 4 is such an example.
- If an implicit, closed form for the envelope ***Γ*** cannot be found, it is still possible to approximate the latter by a mesh of (*N* − 1)-dimensional simplices (i.e., triangles, tetrahedra, and so on), to arbitrary precision. The simplicial mesh should extend from the (*N* − 2)-dimensional curve where the envelope is tangent to the threshold, Equation (14), to the one where it is tangent to the backpropagated threshold at *t* = *h*, Equation (15). In this case instead of an inequality χ(***s***) < 0 we have a system of linear inequalities.
  Exact spike detection can still be achieved in this case. Owing to the concavity of the envelope discussed in the previous section, all the simplices of the mesh lie in the no-spike region. Therefore, if the system of linear inequalities is satisfied the state surely lies in the no-spike region. If the system is not satisfied, a spike can be assumed to occur, and the threshold-crossing time can be sought by a root-finding algorithm. In this case there still is a small chance that the state lies in one of the small no-spike pockets between the envelope and the mesh, but the absence of a spike would still be detected by noting that the root-finding algorithm has no solution in this case.
  The coordinates of the vertices of the mesh are obtained via Equation (21) by selecting a set of points {(***y***_*i*_, *t*_*i*_)}, 0 < *t*_*i*_ < *h*. Since the envelope is a ruled surface, as shown in section 3.2.2, the mesh can be conveniently chosen in such a way that one face of each simplex fully lies on the envelope. The coefficients of the linear inequalities related to the mesh therefore do not depend on the state of the neuron, but depend on the parameters of the model and, in exponential form, on the timestep *h*. Their functional form needs to be determined *only once* for a given model.
  Although less efficient than the case in which an implicit closed-form for the envelope exists, the approximation with a simplicial mesh can still be quite effective, because not all the linear inequalities of the linear system will have to be checked in general: as soon as one of them is not satisfied the test will stop. The most efficient coarseness of the mesh has to be evaluated case-by-case for each model.

Whether an implicit closed form for the envelope or only an approximating mesh is available, the spike test can in any case be optimized by first testing a set of linear inequalities that delimit the larger parts of the spike and no-spike regions. Such set would be a very coarse mesh approximating the envelope. A two-dimensional example is given in **Figure 6**, and we discuss this with a concrete example in section 4.4.

In the following we denote by χ(***s***) < 0 either the closed form of the inequality, if it exists, or the system of linear inequalities that approximate it, if the former does not exist in closed form.

#### 3.2.4. Boundary intersections and final system

If a state does not cross the threshold hyperplane at any time *t* ∈ [0, *h*], then it must, by definition, belong for every *t* to the image of the half-space that lies below threshold, when this half-space is backpropagated by the time *t*. Mathematically, this is simply the statement of the equivalence

(24) $$\begin{array}{l} k^{⊺}\left(Ms+m\right)<\kappa \Leftrightarrow \left(k^{⊺}M\right)s<\left(\kappa -k^{⊺}m\right) \end{array}$$

established in section 3.1, where ***M*** and ***m*** are the coefficients of the affine evolution by time *t*.

By construction in the previous subsection, the inequality χ(***I***, *V*) < 0 defines the region of intersection of all such backpropagated half-spaces, bounded by the envelope χ(***I***, *V*) = 0. We just need to join to it the condition for the boundaries corresponding to the times *t* = 0 and *t* = *h*, *V* < θ, $k_{h}^{⊺}s<\kappa_{h}$, discussed in section 3.2.1.

The states that do not cross the threshold during the time interval [0, *h*] belong therefore to the no-spike region defined, in coordinates, by

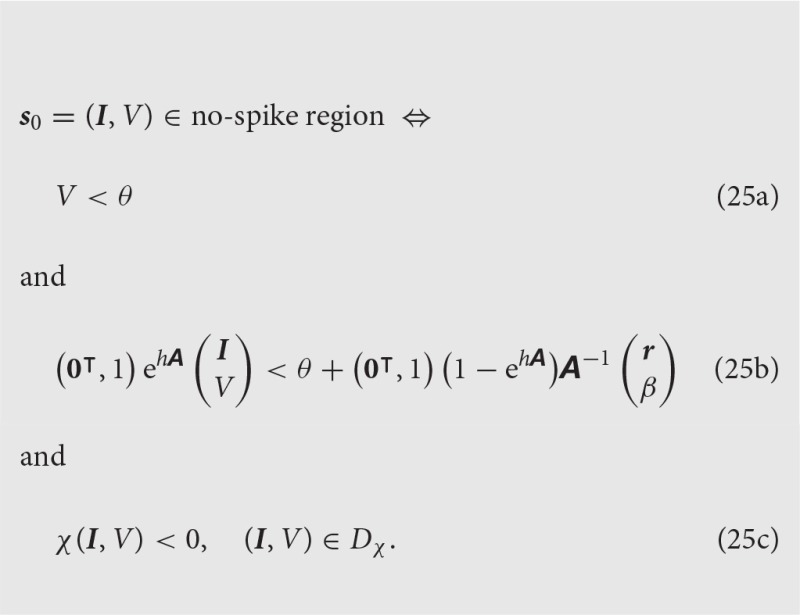



Some inequalities in this system may turn out to be redundant, i.e., automatically satisfied if the remaining ones are, and can thus be dropped. Section 4.2.3 illustrates such a redundancy.

### 3.3. The region of missed spikes

In the previous section we mentioned that the *N*-dimensional product manifold formed by the (*N* − 1)-dimensional threshold hyperplane and the time interval [0, *h*] presents self-intersections when mapped to the state-space. Two-dimensional examples of such a region are shown in Figure 3, lower panel, and in **Figure 6**, the region called *S*_2_.

A state ***s*** in the self-intersection region corresponds to two or more different coordinates (***x***, *t*):

(26) $$\begin{array}{l} s=E\left(x,t\right)=E\left(x^{\prime },t^{\prime }\right),\;t\neq t^{\prime }. \end{array}$$

Note that ***x*** ≠ ***x***′, *t* = *t*′ is impossible for an affine transformation, since the first column of Equation (16) can never vanish. The condition above simply means that ***s*** crosses the threshold at ***x*** after an interval *t* and at ***x***′ after an interval *t*′. By continuity of the trajectory of Equation (7), one of the two must be a crossing from above, and one from below: this is exactly the scenario of double threshold crossing illustrated in Figure 1.

Suppose we have a probability distribution for the initial states, for example one that is invariant under the dynamical law (Equation 7). The probability of the self-intersection region *P*(*X*) is the probability that the initial state will lead to a double-crossing of the threshold, and therefore be missed. This fact will be used in section 4.4.2 to estimate the number of spikes missed.

## 4. Implementation example: leaky integrate-and-fire neuron with exponentially decaying post-synaptic currents

### 4.1. The example model: terms in block form

In the previous section we mathematically developed the idea of propagating the threshold backward in time in order to check whether a threshold-crossing occurs in a time-stepped dynamical process. The derivation, valid for an affine subthreshold dynamical law, is general and therefore also quite abstract; moreover, it involves a couple of mathematical steps (III and IV in section 2) for which no general formula can be given.

To explain the idea in more concreteness and to give an example of how to face all its steps, we now apply the scheme to a simple but relevant model with a 2-dimensional state-space: the leaky integrate-and-fire neuron with exponentially decaying post-synaptic currents. This model satisfies a homogeneous linear dynamical law on a 3-dimensional state-space (Rotter and Diesmann, 1999), where the third coordinate is the input current. If this current is constant, the dynamical law can be rewritten as a 2-dimensional affine one.

Leaky integrate-and-fire models, despite their simplicity, approximate the behavior of real neurons with high accuracy (Rauch et al., 2003). The model with exponential synaptic currents captures important properties of real neurons: The post-synaptic potential has a finite rise and decay time and the membrane potential is a continuous function of time. Continuity avoids artificial synchronization, present in simpler models. Moreover, the model is to some extent analytically tractable. For short synaptic time constants, the mean firing rate (Fourcaud and Brunel, 2002) as well as the linear response to small inputs (Schucker et al., 2015) can be obtained analytically.

The example model has a 2-dimensional state-space for a single neuron, defined by the post-synaptic current *I* and the membrane potential *V*, which are also our coordinates. Its subthreshold interspike dynamical law (a) in section 3 is affine:

(27a) $$\begin{array}{l} \begin{array}{cc} İ\left(t\right) & =-\frac{1}{\tau_{\text{s}}}I\left(t\right),\\ \dot{V}\left(t\right) & =\frac{1}{C}I\left(t\right)-\frac{1}{\tau }V\left(t\right)+\frac{1}{C}I_{\text{e}}, \end{array} \end{array}$$

or in matrix form

(27b) $$\begin{array}{ll} \frac{\text{d}}{\text{d}t}\left(\begin{array}{c} I\\ V \end{array}\right) & =\left(\begin{array}{cc} -\frac{1}{\tau_{\text{s}}} & 0\\ \frac{1}{C} & -\frac{1}{\tau } \end{array}\right)\left(\begin{array}{c} I\\ V \end{array}\right)+\left(\begin{array}{c} 0\\ \frac{1}{C}I_{\text{e}} \end{array}\right), \end{array}$$

where *C* is the membrane capacitance, τ is the membrane time constant, *I* is the synaptic input current, and *I*_e_ is the external input current. The membrane potential *V* is subject to dissipation with time constant τ and integrates the post-synaptic current *I*. The latter decays exponentially with time constant τ_s_. Typical values of the parameters are τ = 10 ms, *C* = 250 pF, τ_s_ = 2 ms, and the threshold θ = 20 mV.

Incoming spikes are incorporated in the equation for the current as Dirac deltas, and the external current *I*_e_ has jump discontinuities in time. In the timestepped evolution, such discontinuous events are implemented as instantaneous changes in the initial state ***s***_0_ at each timestep. Hence we do not need to consider them explicitly in the equations above (Rotter and Diesmann, 1999).

In terms of the block form of section 3.1 we have

(28) $$\begin{array}{l} \begin{array}{ccc} B=\left(-\frac{1}{\tau_{\text{s}}}\right), & d=\left(0\right), & r=\left(0\right),\\ c^{⊺}=\left(\frac{1}{C}\right), & \alpha =-1/\tau , & \beta =\frac{1}{C}I_{\text{e}}. \end{array} \end{array}$$

The exponential of ***A*** is

(29) $$\exp(-tA)=\left(\begin{array}{cc} e^{\frac{t}{\tau_{\text{s}}}} & \text{o}\\ \frac{\left(e^{\frac{t}{\tau }}-e^{\frac{t}{\tau_{\text{s}}}}\right)\tau \tau_{\text{s}}}{C(\tau -\tau_{\text{s}})} & e^{\frac{t}{\tau }} \end{array}\right),$$

which determines the evolution of the neuron state ***s***_0_ by an affine map as in Equation (8).

Since the state space is 2-dimensional, the “hyperplanes” and “hypersurfaces” of section 3 are straight lines and curves. In particular, the threshold hyperplane is a line; when propagated by a time *t* it maps onto a line with covector and affine term given by Equation (33), explicitly

(30) $$\begin{array}{l} \begin{array}{l} k_{t}^{⊺}=\left(\begin{array}{c} \frac{\left({\text{e}}^{-\frac{t}{\tau }}-{\text{e}}^{-\frac{t}{\tau_{\text{s}}}}\right)\tau \tau_{\text{s}}}{C\left(\tau -\tau_{\text{s}}\right)}e^{-\frac{t}{\tau }} \end{array}\right),\\ \kappa_{t}=\theta -\frac{I_{\text{e}}\tau \left(1-{\text{e}}^{-\frac{t}{\tau }}\right)}{C}. \end{array} \end{array}$$

For this model, testing for threshold-crossing by checking the intersection of a propagated state and the threshold line means finding the roots of this equation in *t*, given the initial state ***s***_0_ = (*I, V*):

(31) $$\begin{array}{l} {\text{e}}^{-\frac{t}{\tau }}V+\frac{I_{\text{e}}\tau }{C}\left(1-{\text{e}}^{-\frac{t}{\tau }}\right)+I\frac{\tau \tau_{\text{s}}}{C}\frac{{\text{e}}^{-\frac{t}{\tau }}-{\text{e}}^{-\frac{t}{\tau_{\text{s}}}}}{\tau -\tau_{\text{s}}}=\theta ,\;0⩽t⩽h, \end{array}$$

for which we cannot find a solution in analytic form.

### 4.2. The threshold-crossing condition

#### 4.2.1. Hypervolume in parametric form

The product manifold “threshold line × time interval” is in this case 2-dimensional, with coordinates (*x, t*). Here ***x*** is the value of the current at threshold crossing and *t* the corresponding time point. Its mapping *E*, Equation (12), to the state-space is

(32) $$\begin{array}{l} E:(x,t)\mapsto (I,V)=({\text{e}}^{-\frac{t}{\tau_{\text{s}}}}x,{\text{ e}}^{-\frac{t}{\tau }}\theta +\frac{I_{\text{e}}\tau }{C}(1-{\text{e}}^{-\frac{t}{\tau }})\\ \;+x\frac{\tau \tau_{\text{s}}}{C}\frac{{\text{e}}^{-\frac{t}{\tau }}-{\text{e}}^{-\frac{t}{\tau_{\text{s}}}}}{\tau -\tau_{\text{s}}}). \end{array}$$

This map is shown in Figure 3: the *x* isocurves are yellow and in the lower panel they represent the trajectories of states terminating on the threshold; the *t* isolines are blue and represent the threshold line propagated at different times. Each area element d***x*** ∧ d*t* in the domain—the small rectangles in the upper plane—is mapped into an area element d***I*** ∧ d*V* in the image. Note how these area elements are rotated and sheared. The thicker green curve is the set of singular points where the Jacobian determinant, i.e., the determinant of the derivative, vanishes: det(*E*′) = 0. Such points are singular because around them the images of the area elements get flattened by one dimension. In section 4.3 we discuss the region of self-intersection bounded by the thick light cyan line, the thick dark blue line, and the thick green curved line.

For fixed *t*, the map ***x*** ↦ *E*(***x***, *t*) is affine, and its image is a hyperplane representing the states on the set of states at threshold propagated backwards in time for an interval *t*. Combining it with Equations (4–6) we find that the set of states of backpropagated threshold state has *t*-dependent normal and affine terms

(33) $$\begin{array}{l} \begin{array}{c} k_{t}^{⊺}=\left(\begin{array}{c} 0^{⊺},1 \end{array}\right){\text{e}}^{tA}\\ \kappa_{t}=\theta +\left(\begin{array}{c} 0^{⊺},1 \end{array}\right)\left(1-{\text{e}}^{tA}\right)A^{-1}\left(\begin{array}{c} r\\ \beta \end{array}\right). \end{array} \end{array}$$

The inequality $k_{t}^{⊺}s<\kappa_{t}$ determines the backpropagated half-space, which is below threshold. An example of the map E for a model with a three-dimensional state space is shown in Figure 4.

#### 4.2.2. Hypervolume boundary in parametric form

The boundaries of the image of the map *E* must be, as explained in section 3.2.3, a subset of the images of the boundaries of the domain, **R** × {0} and **R** × {*h*}, and the envelope.

The images of the boundaries, with general Equations (14, 15), in terms of (*I, V*) follow

(34) $$\begin{array}{l} V=\theta , \end{array}$$

(35) $$\begin{array}{l} V={\text{e}}^{\frac{h}{\tau }}\theta +\frac{I_{\text{e}}\tau }{C}\left(1-{\text{e}}^{\frac{h}{\tau }}\right)+\frac{I\tau }{C}\tau_{\text{s}}{\text{e}}^{-\frac{h}{\tau_{\text{s}}}}\frac{{\text{e}}^{\frac{h}{\tau }}-{\text{e}}^{\frac{h}{\tau_{\text{s}}}}}{\tau -\tau_{\text{s}}}. \end{array}$$

The set of critical points of the map *E* is in this case a 1-dimensional curve, given in parametric form by

(36) $$\begin{array}{l} \Gamma :t\mapsto \left({\text{e}}^{\frac{t}{\tau_{\text{s}}}}\left(\frac{\theta C}{\tau }-I_{\text{e}}\right),\;\frac{\tau {\text{e}}^{\frac{t}{\tau }}-\tau_{\text{s}}{\text{e}}^{\frac{t}{\tau_{\text{s}}}}}{\tau -\tau_{\text{s}}}\frac{\tau }{C}\left(\frac{\theta C}{\tau }-I_{\text{e}}\right)+\frac{\tau }{C}I_{\text{e}}\right); \end{array}$$

the coordinate ***y*** of the general form (Equation 21) do not exist in this case, because the threshold is 1-dimensional. Figure 3 visualizes the set by the green curve.

#### 4.2.3. Hypervolume boundary in implicit form

The next step in our procedure is to convert the parametric Equation (36) of the envelope into an explicit or implicit equation for the coordinates (*I, V*). As section 3.2.3 does not provide a general algorithm, below we illustrate the process using our example model.

By equating the first component of Equation (36) to the coordinate *I* and solving for *t*, we find

(37a) $$\begin{array}{l} t=-\tau_{\text{s}}\ln\;\left[\left(\frac{\theta C}{\tau }-I_{\text{e}}\right)/I\right], \end{array}$$

subject to the condition for *I*

(37b) $$\begin{array}{l} {\text{e}}^{-\frac{h}{\tau_{\text{s}}}}⩽\frac{1}{I}\left(\frac{\theta C}{\tau }-I_{\text{e}}\right)⩽1, \end{array}$$

required for 0 ⩽ *t* ⩽ *h* and a real logarithm.

Substituting Equation (37a) into the *V* coordinate of Equation (36) we find

(38) $$\begin{array}{l} V=\frac{\tau I_{\text{e}}}{C}+\frac{\tau I}{C}\frac{\tau {\left[\left(\frac{\theta C}{\tau }-I_{\text{e}}\right)/I\right]}^{1-\frac{\tau_{\text{s}}}{\tau }}-\tau_{\text{s}}}{\tau -\tau_{\text{s}}} \end{array}$$

subject to the condition (Equation 37b). Let us analyse this equation, in view of its extension to an inequality of the form χ(*I, V*) < 0 as required in section 3.2.3. First, we observe that

(39) $$\begin{array}{l} \frac{\tau {\text{e}}^{\frac{t}{\tau }}-\tau_{\text{s}}{\text{e}}^{\frac{t}{\tau_{\text{s}}}}}{\tau -\tau_{\text{s}}}⩽1\text{ for }0⩽t⩽h. \end{array}$$

The inequality can be proven by studying the derivative of the fraction with respect to *t*. The derivative is always negative in the range above and the only maximum of the fraction is the value unity assumed at *t* = 0.

Inspection of Equation (38) and of its parametric form (Equation 36) shows that we must consider three cases: *I*_e_ ⋛ θ*C*/τ, i.e., whether the external current is smaller or larger than the *rheobase current*

(40) $$\begin{array}{l} I_{\theta }=\theta C/\tau ; \end{array}$$

this is the current necessary to reach threshold in an infinite time starting from any state with *I* ⩽ 0.

• If *I*_e_ < *I*_θ_, then *I* is restricted to

(41) $$\begin{array}{l} 0<I_{\theta }-I_{\text{e}}⩽I⩽{\text{e}}^{\frac{h}{\tau_{\text{s}}}}\left(I_{\theta }-I_{\text{e}}\right). \end{array}$$

In this case, using the inequality (Equation 39), the *V* component of the envelope (Equation 36) is always smaller than the threshold:

(42) $$\begin{array}{l} V\equiv \frac{\tau I_{\text{e}}}{C}+\frac{\tau I}{C}\frac{\tau {\left[\left(I_{\theta }-I_{\text{e}}\right)/I\right]}^{1-\frac{\tau_{\text{s}}}{\tau }}-\tau_{\text{s}}}{\tau -\tau_{\text{s}}}⩽\theta \text{ when }I_{\text{e}}<I_{\theta }. \end{array}$$

• If *I*_e_ > *I*_θ_, then *I* must be negative and restricted to

(43) $$\begin{array}{l} {\text{e}}^{\frac{h}{\tau_{\text{s}}}}\left(I_{\theta }-I_{\text{e}}\right)⩽I⩽I_{\theta }-I_{\text{e}}<0. \end{array}$$

In this case, using the inequality (Equation 39), the *V* component of the envelope (Equation 36) is always larger than the threshold:

(44) $$\begin{array}{l} V\equiv \frac{\tau I_{\text{e}}}{C}+\frac{\tau I}{C}\frac{\tau {\left[\left(I_{\theta }-I_{\text{e}}\right)/I\right]}^{1-\frac{\tau_{\text{s}}}{\tau }}-\tau_{\text{s}}}{\tau -\tau_{\text{s}}}⩾\theta \text{ when }I_{\text{e}}>I_{\theta }. \end{array}$$

• If *I*_e_ = *I*_θ_, the envelope degenerates to a point: **Γ**(*t*) = (0, θ), which is the limit point reached in infinite time from any initial point in the no-spike region; this is the geometric interpretation of the equality of external and rheobase currents.

The function representing the envelope, Equation (23) of section 3.2.3, has in this case the explicit form

(45) $$\begin{array}{ll} \chi \left(I,V\right) & :=V-\frac{\tau I_{\text{e}}}{C}-\frac{\tau I}{C}\frac{\tau {\left[\left(I_{\theta }-I_{\text{e}}\right)/I\right]}^{1-\frac{\tau_{\text{s}}}{\tau }}-\tau_{\text{s}}}{\tau -\tau_{\text{s}}},\text{with }\left(I,V\right)\in D_{\chi }:=\left[I_{\theta }-I_{\text{e}},{\text{e}}^{\frac{h}{\tau_{\text{s}}}}\left(I_{\theta }-I_{\text{e}}\right)\right]\times \text{R}. \end{array}$$

If we calculate its differential, as discussed in section 3.2.3, we find that the latter is a positive multiple of the differential of the backpropagated threshold for each *t*. This is true in each of the three cases above. Consequently, the inequality χ(*I, V*) < 0, (*I, V*) ∈ *D*_χ_ always includes the no-spike region.

In summary, we arrive at an analytic, implicit equation for the curved boundary (Equation 45). The expression is a transcendental function in *I*, owing to the generally irrational exponent 1 − τ_s_/τ, but it is used in an inequality, hence we do not need to find its roots.

#### 4.2.4. Boundary intersections and final system

We can now assemble the system of inequalities defining the no-spike region consisting of the boundaries (Equations 34, 35), and the envelope (Equation 45).

With some rearrangements, simplifications, and the introduction of two new functions *f*, *b*, the condition reads:

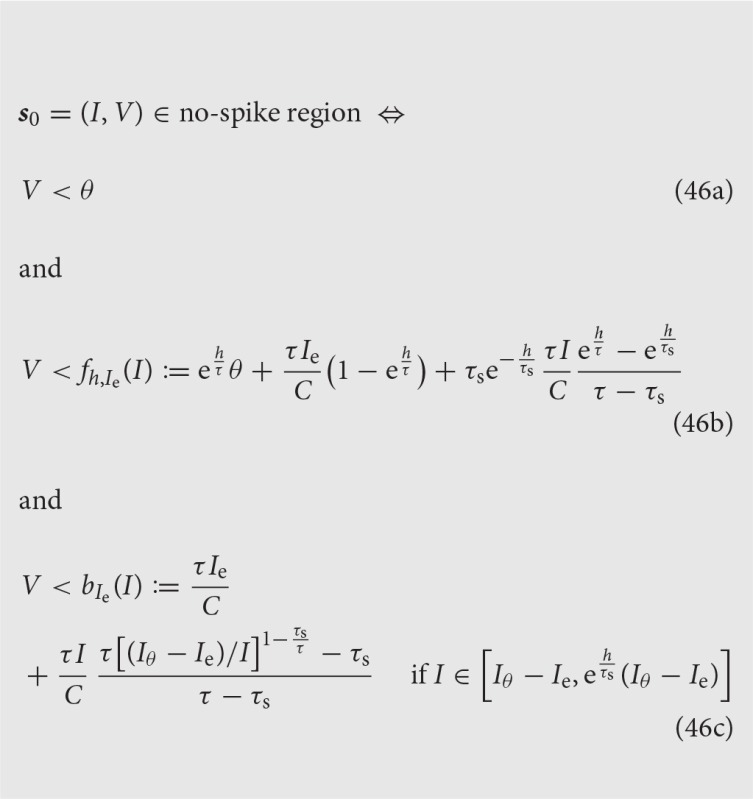



**Figure 5:**
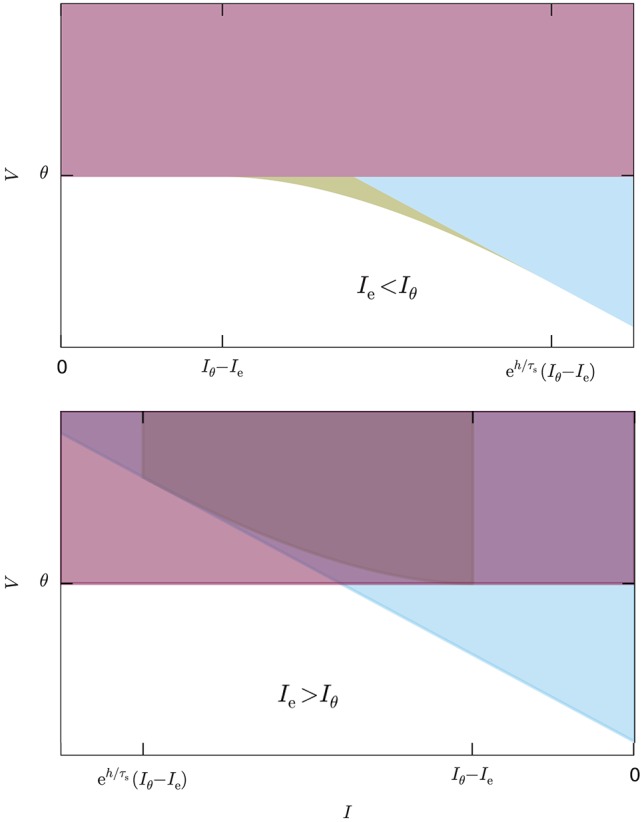
System of inequalities, Equation (46), determining the no-spike region. The colored areas represent the complementary inequalities of the system (46), so the solution of that system is the white area. The red region delimited by the horizontal line corresponds to the first equation of the system, the blue region delimited by the inclined line to the second, and the yellow region delimited by the curved line to the third. **Upper:** Case *I*_e_ < *I*_θ_, all inequalities necessary. **Lower:** Case *I*_e_ > *I*_θ_, the third inequality is redundant.

Figure 5 illustrates the system for the two cases *I*_e_ < *I*_θ_ and *I*_e_ > *I*_θ_. In the former case all three inequalities are necessary; in the latter, as well as for *I*_e_ = *I*_θ_, the last inequality is automatically enforced by the first because its right-hand side is larger than the threshold θ [see Equation (44)].

### 4.3. The region of missed spikes

In Figure 3, lower panel, the trajectories of several states during a timestep *h* and ending on the threshold line are represented by yellow curves. In that figure we can identify a region were such trajectories self-intersect: it is bounded by a segment of the thick light blue line, a segment of the thick dark blue line, and a portion of the thick green curved line. Trajectories with initial states in this region must therefore cross the threshold twice during the interval ]*t*_0_, *t*_0_ + *h*]. As explained in section 3.3, their threshold-crossing is not detected by the sole condition *V*[***s***(*t*+*h*)] ⩾ θ. All states in this region thus generate spikes that are missed by the standard test (Equation 1).

This region is crucial for the comparison of the performances of schemes implementing the present lossless method and schemes relying on the standard test (Equation 1). This comparison is quantitatively made in section 4.4.3.

### 4.4. Optimization, time performance, and accuracy

#### 4.4.1. Numerical implementation and optimization

In the last section we arrived at the system of three inequalities (Equation 46) that determines whether or not the current state (*V, I*) will cross the threshold within the timestep *h*. The state will cross the threshold if the system is not satisfied, and it will not cross the threshold if the system is satisfied. This system of inequalities constitutes the lossless method in the present model. The system requires the current timestep *h*, state (*V, I*), and external electric current *I*_e_ as inputs (cf. section 3). The timestep *h* is the minimum between the global timestep and the time interval up to the next input coming from other neurons, hence it can differ every time the test is called. The external electric current *I*_e_ may also vary, stepwise, during the simulation, hence it may also be distinct at each call of the test.

If the system of inequalities is satisfied, thus predicting the absence of a spike within a timestep *h*, then the state of the neuron is evolved by applying the propagator (Equation 8) with Equations (28, 29), leading to a new state, and the procedure starts again. If the system is not satisfied, thus predicting the occurrence of a spike within *h*, it is then necessary to compute the time *t*_θ_ at which the threshold is crossed. This calculation, explained in Appendix section A.1, is made by interpolation between the current state and time, and the state and time *t*_max_ at which the membrane potential would reach its maximum if allowed to increase above threshold. Once *t*_θ_ is calculated, a spike is emitted, communicated to the post-synaptic neurons, and the membrane potential is reset to *V*_reset_ for a refractory period. When this refractory period is over the procedure starts again.

In the evolution loop just described, the membrane potential is reset to a value below threshold as soon as it crosses the latter. Thus no initial state can have *V* ⩾ θ. This means that the first inequality (Equation 46a) in the system is always satisfied and can be dropped. Only inequalities (Equations 46b,c) have to be assessed, leading to the reduced system

(47) $$\begin{array}{l} \{\begin{array}{lll} V & < & f_{h,I_{\text{e}}}(I),\\ V & < & b_{I_{\text{e}}}(I). \end{array} \end{array}$$

We first discuss the case *I*_e_ < *I*_θ_ ≡ θ*C*/τ.

**Figure 6:**
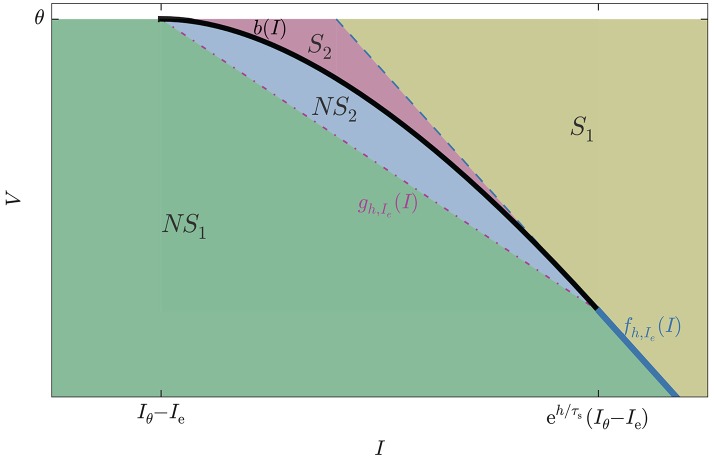
State-space subregions formed by the intersections of the reduced inequalities (Equation 47): *V* < *f*_*h*,*I*_e__(*I*) (straight blue line, partly dashed partly continuous) and *V* < *b*_*h*_(*I*) (black curve), and by the auxiliary inequality (Equation 49): *V* < *g*_*h*,*I*_e__(*I*) (dot-dashed straight purple line). The spike region is *S*_1_ ∪ *S*_2_, the no-spike region is *NS*_1_ ∪ *NS*_2_. The subregion *S*_2_ contains all states that emit spikes undetected by the standard test (Equation 1); they are detected by the lossless method.

The geometric meaning of the inequalities above is illustrated in Figure 6. The figure shows four regions: *NS*_1_, *NS*_2_, *S*_1_, *S*_2_. The no-spike region is the union of *NS*_1_ and *NS*_2_, the spike region the union of *S*_1_ and *S*_2_. In the figure they are separated by a thick line, partly curved and black, partly straight and blue. Subregion *S*_2_ is particularly important: it is the region of missed spikes discussed in section 4.3, corresponding to the self-intersection region of Figure 3, lower panel. It contains those states that lead to spikes missed by schemes that rely on the standard test (Equation 1).

Region *S*_1_ is separated from *S*_2_ by a dashed blue line, and from *NS*_1_ by a continuous blue line, the continuation of the dashed one. This partly dashed, partly continuous blue line corresponds to the equation *V* = *f*_*h*,*I*_e__(*I*). Hence if the inequality *V* < *f*_*h*,*I*_e__(*I*) is *not* satisfied then the initial state is in region *S*_2_ or on its blue boundary, and there will be a spike. If the inequality is satisfied the state could be in *S*_2_ − spike − or *NS*_1_ ∪ *NS*_2_ − no spike; an undetermined case. This inequality requires modest computational costs because it is linear in *I* and *I*_e_ and involves exponentials of *h*. Regions *S*_2_ and *NS*_2_ are separated by a black curve: this is the envelope, corresponding to the equation *V* = *b*_*I*_e__(*I*) for $I\in \left[I_{\theta }-I_{\text{e}},{\text{e}}^{\frac{h}{\tau_{\text{s}}}}\left(I_{\theta }-I_{\text{e}}\right)\right]$. Hence if the inequality *V* < *b*_*I*_e__(*I*) is *not* satisfied the initial state is in *S*_2_ or on its boundary, and there will be a spike. If the inequality is satisfied the initial state is either in *NS*_2_, or in *NS*_1_ with $I_{\theta }-I_{\text{e}}<I<{\text{e}}^{\frac{h}{\tau_{\text{s}}}}\left(I_{\theta }-I_{\text{e}}\right)$, and no spike will occur.

The computationally most expensive inequality is *V* < *b*_*I*_e__(*I*) because it involves irrational powers of *I* and *I*_e_. It is advisable to avoid its direct computation as often as possible by pre-testing a linear inequality. In section 3.2.3 we discussed how such a pre-test is indeed possible thanks to the convexity of the no-spike region. There, we argued that the curved envelope can be approximated by a simplicial mesh, which simply reduce to one straight segment in the present two-dimensional case: this is the dot-dashed red line in Figure 6, separating *NS*_1_ and *NS*_2_. This line has equation *V* = *g*_*h*,*I*_e__(*I*) with

(48) $$\begin{array}{l} g_{h,I_{\text{e}}}\left(I\right):=\theta +\frac{\tau_{\text{s}}{\text{e}}^{\frac{h}{\tau_{\text{s}}}}}{\tau -\tau_{\text{s}}}\frac{\tau }{C}I+{\text{e}}^{\frac{h}{\tau }}\frac{\tau }{C}\left(I_{\theta }-I_{\text{e}}\right), \end{array}$$

and the corresponding inequality

(49) $$\begin{array}{l} V<g_{h,I_{\text{e}}}\left(I\right) \end{array}$$

has the same computational costs as *V* < *f*_*h*,*I*_e__(*I*).

If the auxiliary inequality *V* < *g*_*h*,*I*_e__(*I*) is satisfied, the initial state is in *NS*_1_ and *V* < *b*_*I*_e__(*I*) is also satisfied. It is therefore convenient to test the auxiliary inequality before the computationally costly one, which can be discarded if the test is positive. Figure 6 suggests that this test might be positive for the majority of initial states because region *NS*_1_ is much wider than *NS*_2_. This possibility would be very advantageous, but we now argue that it should be verified by a dynamical analysis.

The system (47) can be translated into a computational algorithm in several different ways, depending on the order of evaluation of its two inequalities and of the auxiliary inequality (49). In simplified terms, such an algorithm consists in a sequence of tests—variously implemented as if, and, or constructs—for finding the initial state in space-time regions *R*_1_, *R*_2_, and so on. The order of these tests is important. The average time cost of the algorithm in a long simulation is given by $\underset{i}{\sum }p_{i}c_{i}$, where *p*_*i*_ is the frequency with which states are found in region *R*_*i*_, which we call “occupation frequency,” and *c*_*i*_ is the cumulative time cost of the test for region *R*_*i*_. This time cost *c*_*i*_ is cumulative in the sense that all tests up to the (*i*−1)th must have been performed, with false outcomes, to arrive at the test for *R*_*i*_. The efficiency of an algorithm therefore depends on the mathematical form of the inequalities defining a region and on the occupation frequencies of the regions, determined by the dynamics. These two factors can be extrapolated by a theoretical analysis, or more practically measured by running long test simulations with typical network setups corresponding to the cases one is interested in.

We now try to determine the most efficient algorithm for the present case. Region *S*_1_ is the least costly, because bounded by one line and therefore involving one inequality linear in *I*; then region *NS*_1_, bounded by two lines involving two linear inequalities; and finally regions *NS*_2_ and *S*_2_, bounded by the curve that involves rational exponentiation. For this example model, we tried different orderings but show here only two possible extreme cases to illustrate that there is no significant difference in the computational cost.

Algorithm 1 is based on the assumption that the occupation frequency of a subregion is proportional to that subregion's relative size. If we check the two largest first, in the order *NS*_1_, *S*_1_, *S*_2_, *NS*_2_, we are therefore more likely to exit the test in its first if branches. The value of *g*_*h*,*I*_e__ is used in two if branches, so it is computed just once and saved before the if sequence in order to save some computations.

**Table:** 

**Algorithm 1**
**bool** is_spike(*h*):
pre-compute *g*_*h*,*I*_e__(*I*)
**if** *V* ⩽ *f*_*h*,*I*_e__(*I*) **and** *V* < *g*_*h*,*I*_e__(*I*) **then**
return **false**
**else if** *V* ⩾ *g*_*h*,*I*_e__(*I*) **then**
return **true**
**else if** *V* ⩾ *b*_*I*_e__(*I*) **then**
return **true**
**else**
return **false**

Algorithm 2 uses a composite or-and condition rather than several ifs. Assuming left-to-right evaluation, the algorithm corresponds to testing first *S*_1_ (left side of or), then *S*_2_ (right side of or), either case leading to a spike. If neither is true, no further tests are necessary because the state must necessarily be in the no-spike region. The test for *S*_2_ is made less costly on average by using the auxiliary inequality (Equation 49). This algorithm uses one test less overall than the previous one, but it may require one more test on average, if *NS*_1_ is the region with highest occupation frequency. The left-to-right evaluation assumption does not always hold in modern processors, which build their own statistics to optimize the test order of logical constructs.

**Table:** 

**Algorithm 2**
**bool** is_spike(*h*):
**if** *V* ⩾ *g*_*h*,*I*_e__(*I*) **or**
*V* ⩾ *f*_*h*,*I*_e__(*I*) **and** *V* ⩾ *b*_*I*_e__(*I*) **then**
return **true**
**else**
return **false**

The analysis assumed *I*_e_ < *I*_θ_ ≡ θ*C*/τ. The reduced system (47) and the Algorithms 1 and 2 are, however, also valid in the case that *I*_e_ ⩾ *I*_θ_, corresponding to the lower panel of Figure 5. In this case subregions *NS*_2_ and *S*_2_ do not exist below the threshold, and the inequalities *V* < *b*_*I*_e__(*I*) and *V* < *g*_*h*,*I*_e__(*I*) are always satisfied when *V* < θ; they always evaluate to true in both algorithms. Both algorithms therefore correctly distinguish spiking from non-spiking states in this case, although they become inefficient owing to the additional superfluous evaluations of *b*_*I*_e__(*I*) and *g*_*h*,*I*_e__(*I*) for spiking states. We decide not to modify them in the present work because the case *I*_e_ ⩾ *I*_θ_ is unusual in real applications. More efficient algorithms for this case can be designed by interested readers following the guidelines just given in this section.

#### 4.4.2. Occupation frequencies

**Figure 7:**
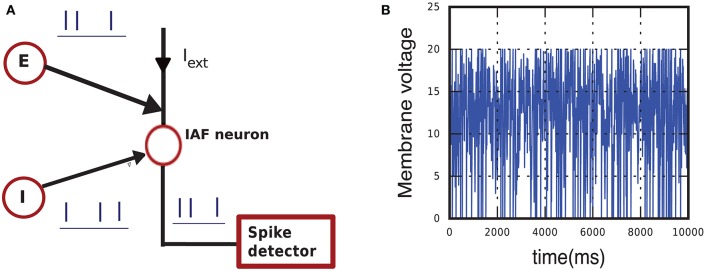
**(A)** Schematic of the simulation setup used to calculate occupation frequencies and to compare the hybrid scheme with lossless method Algorithm 1, with lossless method 2, and with the standard test. The neuron model (empty red circle), implementing one of the three schemes, receives input from an external current and from one excitatory (E) and one inhibitory (I) Poisson generator. The total input has mean μ and variance σ^2^. **(B)** Sample of membrane-potential evolution for μ = 15 mV and σ^2^ = 25 mV^2^.

We want to measure the occupation frequencies in the typical case of a neuron embedded in a recurrent network, receiving fluctuating synaptic input. The setup for this simulation is illustrated in Figure 7A and its formula explained in Appendix section A.2. One neuron is coupled with strengths *J* and −*J* to an excitatory and an inhibitory Poisson generator and also receives a constant external current. The Poisson generators each mimic an excitatory and an inhibitory population. The neuron thus receives a fluctuating input current having average μ and variance σ^2^. In this instance the code of the simulation includes a subroutine that informs us of the current state every time the lossless method test is called, without altering the test or the dynamics.

**Figure 8:**
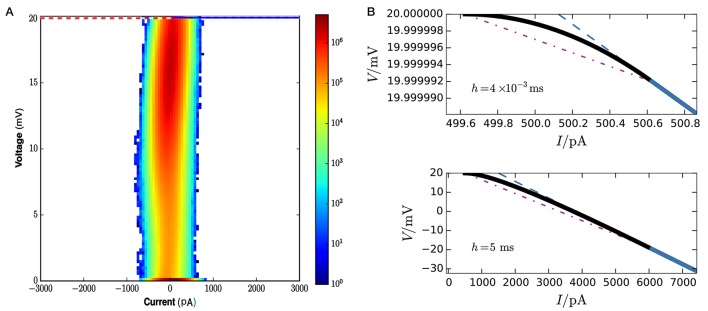
**(A)** Frequency density of states over state space at each call of the threshold-crossing test, for network parameters μ = 15 mV, σ^2^ = 25 mV^2^, *J* = 0.1 mV, excitatory and inhibitory time constants, τ_exc_ = τ_inh_ = 2 ms. The colorbar is in units of 6 × 10^9^ mV^−1^pA^−1^ (obtained from total number of events × area element). The dotted purple line is the threshold θ. The only visible region in this plot is *NS*_1_. and the horizontal blue line is the boundary between no-spike and spike regions. The spike region *S*_1_ ∪ *S*_2_ and subregion *NS*_2_ are not visible on this scale because of the exceedingly small average timestep *h* = 4 × 10^−3^ ms. To discern them we need to zoom in, as done in upper panel **(B)**: the curved envelope and the two straight lines that separate *S*_1_, *S*_2_, and *NS*_2_ extend horizontally and vertically for just about 1pA and 10^−5^ mV. **(B)** Lower panel. In contrast, for a much larger timestep *h* = 5 ms the three boundaries would have a larger extension, about 5, 500pA and 20 mV, and be discernible in plot **(A)**.

Figure 8A gives a visual idea of the occupation frequencies for μ = 15 mV, σ^2^ = 25 mV^2^, and *J* = 0.1 mV (all expressed in volts through multiplication by a resistance of τ/*C* = 40MΩ), which correspond to the case *I*_e_ < *I*_θ_. These values correspond to a composite average input of 250, 000spikes/s, and a total average input current of 400pA. This presynaptic input makes the neuron fire at an average rate of seven spikes/s. A sample of its membrane evolution is shown in Figure 7B. Subregion *NS*_1_ has the overwhelmingly largest occupation frequency. The other three subregions have actually very small areas, as clear from the axis ranges of Figure 8B, owing to the very small value of the average timestep, *h* = 4 × 10^−3^ ms, given by the inverse of the input rate in this hybrid scheme.

**Figure 9:**
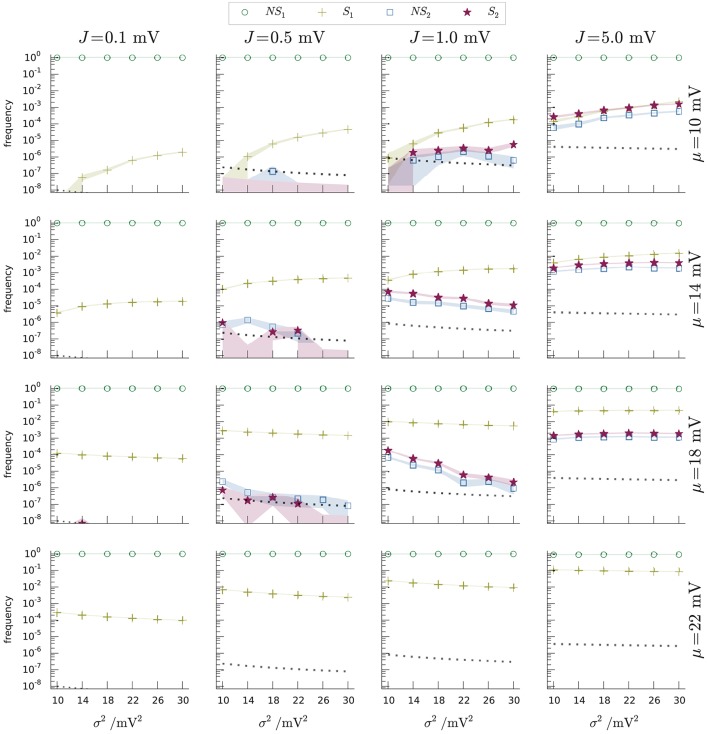
Occupation frequencies of the four subregions *NS*_1_ (green circles), *S*_1_ (yellow crosses), *NS*_2_ (blue squares), *S*_2_ (red stars) of Figure 6, for various sets (μ, σ^2^, *J*) of input-current mean and variance, and synaptic strength. Each panel shows the frequencies vs. current variance for fixed current mean and synaptic strength. The columns have the same *J*, ranging from 0.1 mV (leftmost) to 5 mV (rightmost). The rows have the same μ, ranging from 10 mV (top) to 22 mV (bottom). The frequencies were measured from *N* samples, depending on (μ, σ^2^, *J*). Excitatory and inhibitory time constants, τ_exc_ = τ_inh_ = 2 ms. The dotted lines in each plot show the inverse number of samples 1/*N* for that network regime. The thickness of the segments connecting the data points equals one standard deviation. The shaded regions show where the limiting frequencies (*N* → ∞) are expected to lie with 87% probability, using a Johnson-Dirichlet model with parameter *k* = 0.05 determined by posterior maximization (Johnson, 1932; Good, 1966; Zabell, 1982, 2005; Bernardo and Smith, 2000, § 3.2.5). The frequencies of region *S*_2_ (red stars) are particularly important: they are the frequencies of spike-misses of the standard test (Equation 1).

A more precise comparison of the occupation frequencies of the four regions *NS*_1_, *NS*_2_, *S*_1_, *S*_2_ is shown in Figure 9 for several combinations of three network parameters, producing different dynamical regimes. The parameters are the average μ, the variance σ^2^ of the input current, and the presynaptic coupling strength *J* (all expressed in volts through multiplication by a resistance of τ/*C* = 40MΩ). The values of the parameters (μ, σ^2^, *J*) include typical realistic cases as well as some extreme cases, like unusually high coupling strengths. Each panel of Figure 9 shows the occupation frequencies for a set of dynamical regimes with constant *J*, β, and several σ^2^. The panels in the last row correspond to the case *I*_e_ ≡ μ*C*/τ ⩾ *I*_θ_ ≡ θ*C*/τ, or μ ⩾ θ, in which subregions *NS*_2_ and *S*_2_ do not exist below threshold.

It is important to remember that the boundary and size of the regions of Figure 6 vary with the timestep *h*, which is a parameter of the simulation scheme, not of the dynamics *per se*. In an event-driven or hybrid scheme, this step varies inversely with the event input rate, which for Poisson input generators is proportional to σ^2^/*J*^2^. As a consequence, the frequencies displayed in Figure 9 are not determined by the Liouville distribution of the dynamical trajectory (Equation 27) alone, but also by the details of the numerical-implementation scheme. The dependence of the boundaries on *h* is illustrated in Figure 8B. As *h* decreases, the line *V* = *f*_*h*,*I*_e__(*I*) and the auxiliary line *V* = *g*_*h*,*I*_e__(*I*) gets closer to the threshold, and the subregions *S*_1_, *S*_2_, *NS*_2_ disappear. This is plausible since in the limit of *h* = 0 we are not evolving the initial state at all. As *h* increases the point of tangency between the envelope *V* = *b*_*I*_e__(*I*) and the line *V* = *f*_*h*,*I*_e__(*I*) moves to increasingly lower voltages and higher currents; subregion *S*_2_ takes over *S*_1_ and subregion *NS*_1_ becomes wider. For typical timestep values of several milliseconds, though, subregions *S*_1_, *S*_2_, *NS*_2_ are still very small.

A rough estimate of the dependence of the areas of the bounded regions *S*_2_ and *NS*_2_ on the parameters (μ, σ^2^, *J*), for μ < θ, can be obtained by looking at Figure 9 and considering that these areas together form a triangle with vertices

(50) $$\begin{array}{l} \left(I_{\theta }-I_{\text{e}},\theta \right),\;\left({\text{e}}^{h/\tau_{\text{s}}}\left(I_{\theta }-I_{\text{e}}\right),b_{I_{\text{e}}}\left[{\text{e}}^{h/\tau_{\text{s}}}\left(I_{\theta }-I_{\text{e}}\right)\right]\right),\;\left(I_{f},\theta \right),\\ \text{ with}I_{f}\text{such that}f_{h,I_{\text{e}}}\left(I_{f}\right)=\theta . \end{array}$$

This triangle has base |*I*_*f*_ − (*I*_θ_ − *I*_e_)| and height $\left|\theta -b_{I_{\text{e}}}\left[{\text{e}}^{h/\tau_{\text{s}}}\left(I_{\theta }-I_{\text{e}}\right)\right]\right|$. Expressing *h* and *I*_e_ in terms of μ and σ^2^ using Equation (A3), where *h* is inversely proportional to the input rate *r*_I_ + *r*_E_, we find

(51) $$\begin{array}{l} \text{areas of }S_{2}\text{ and }NS_{2}\propto \frac{C}{\tau }{\left(\theta -\mu \right)}^{2}\left[1-\frac{\tau {\text{e}}^{h/\tau }-\tau_{\text{s}}{\text{e}}^{h/\tau_{\text{s}}}}{\tau -\tau_{\text{s}}}\right]\\ \;\left[\frac{{\text{e}}^{h/\tau_{\text{s}}}\left(\tau -\tau_{\text{s}}\right)\left(1-{\text{e}}^{h/\tau }\right)}{\tau_{\text{s}}\left({\text{e}}^{h/\tau }-{\text{e}}^{h/\tau_{\text{s}}}\right)}-1\right]\text{ with }h\text{=}\frac{\tau J^{2}}{\sigma^{2}}. \end{array}$$

When τ_s_ ≲ τ and *J*^2^ ≲ σ^2^ a Taylor expansion in *J*^2^/σ^2^ to fourth order gives a good approximation, with a relative error below 10%:

(52) $$\begin{array}{l} \text{areas of }S_{\text{2}}\text{ and }NS_{\text{2}}\propto \frac{C}{\tau }{\left(\theta -\mu \right)}^{2}[\frac{\tau^{2}}{4\tau_{\text{s}}^{2}}{\left(\frac{J^{2}}{\sigma^{2}}\right)}^{3}\\ \;+\frac{\tau^{2}(\tau +\tau_{\text{s}})}{{8\tau_{s}}^{3}}{\left(\frac{J^{2}}{\sigma^{2}}\right)}^{4}+\text{O}{\left(\frac{J^{2}}{\sigma^{2}}\right)}^{5}]. \end{array}$$

This approximate formula shows that subregions *NS*_2_ and *S*_2_ grow with the square of the input mean μ and with the third or fourth power of the ratio *J*^2^/σ^2^. Recall that these regions do not exist for μ ⩾ θ. The occupation frequencies do not depend on the areas alone, however, but also on the dynamics, as explained in the previous section. We can identify several other dynamical mechanisms for their dependence on the parameters (μ, σ^2^, *J*):

- an increase in mean input μ leads to more frequent threshold crossings, thus frequently bringing the voltage to its reset value, underneath subregions *NS*_2_ and *S*_2_. The occupation frequencies of these subregions may therefore decrease with μ even though their areas grow with μ;
- for low mean input μ, an increase in variance σ^2^ means a higher chance of high-*V* regions, and thus an increase in the occupation frequencies of *NS*_2_ and *S*_2_, even though their areas shrink with σ^2^;
- for mean input μ close to the threshold, an increase in the variance σ^2^ leads to more frequent threshold crossings, and may thus increase occupation frequency of *S*_2_ with σ^2^, even though its area shrinks with σ^2^.

The occupation frequency of subregion *NS*_1_ (green circles) dominates all others, varying from 90% to 100% depending on the network parameters. Subregion *S*_1_ (yellow crosses) follows in order of frequency and is the most frequently visited between the two spike subregions. Subregions *NS*_2_ (blue squares) and *S*_2_ (red stars) are scarcely visited for lower synaptic amplitudes, with frequencies from 0 to 10^−6^; and slightly more often at higher synaptic amplitudes (frequencies from 0 to 10^−2^).

#### 4.4.3. Time performance and accuracy of a hybrid scheme based on the lossless method

The average time costs of Algorithms 1 and 2 within a hybrid scheme can be assessed by real-time simulation measurements. The average time cost of a hybrid scheme based on the standard test (1) can also be assessed in the same way for comparison. We therefore compare the two algorithms of the lossless method and the standard test in this section.

The basic setup is the same as for the frequency analysis of section section 4.4.1, explained in Appendix section A.2, with network parameters (μ, σ^2^, *J*). The only difference is that in the present case the code does not include the subroutine that informs us of the frequencies, which would otherwise increase and bias the real-time durations of the simulations. Three instances of the basic setup are prepared: in the first the neuron is modeled by a hybrid scheme with lossless method Algorithm 1, in the second the neuron is modeled by a hybrid scheme with lossless method Algorithm 2, and in the third the neuron is modeled by a hybrid scheme with the standard threshold-crossing test (Equation 1). The various random-number-generator seeds of the three instances are exactly the same, so that the three neurons receive exactly the same input, spike-for-spike; this is essential for a fair comparison between the three schemes. The three instances are run for a long time (2 × 10^6^ ms) and repeated (in parallel) for several times (Equation 10), enough to collect reliable statistics. The statistics are collected for the same sets of (μ, σ^2^, *J*) values as in the frequency analysis. The total real-time length of a simulation depends, in all three schemes, on how often the checkpoints and threshold-crossing tests occurs, and this in turn depends on the presynaptic input frequency, as already discussed.

**Figure 10:**
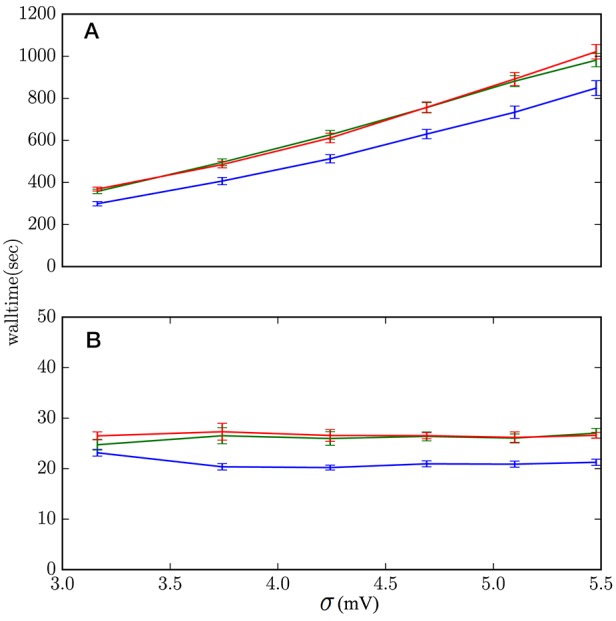
Computational costs of the hybrid scheme with lossless method, Algorithm 1 (red), lossless method, Algorithm 2 (green), and standard threshold-crossing test (1) (blue). **(A)** μ = 18 mV, *J* = 0.1 mV describes regimes where the lossless method has the highest increase in computational cost, around 30%. These are regimes of high activity and frequently incoming spikes. **(B)** μ = 10 mV, *J* = 5 mV describe regimes where the lossless method has the lowest increase in computational cost, around 20%. These are regimes of low activity and infrequently incoming spikes. The data comes from 10 simulations of 2 × 10^6^ ms simulation-time each.

Rather than showing the results for all sets of parameters (μ, σ^2^, *J*), which in this case are not very informative, we show in Figure 10 those with the (*J*, μ) values that yield the slowest and fastest performances. The average computation costs of Algorithms 1 and 2, which embody the lossless method, turn out to be very similar—within each other's standard deviations—and basically identical in comparison with the cost of the scheme based on the standard test (Equation 1). Both are slower than the hybrid scheme with the standard test: from around 30% slower in the case of high-activity regime with frequent incoming spikes (μ = 18 mV, *J* = 0.1 mV), to around 20% slower in the case of low activity regime and infrequent incoming spikes (μ = 10 mV, *J* = 5 mV). These are the extremes shown in Figure 10. In most other sets of network parameters the hybrid scheme with lossless method was around 20–25% slower than the standard hybrid scheme with threshold-crossing test (Equation 1).

As mentioned in section 4.3, subregion *S*_2_ contains all states for which the standard threshold-crossing test misses a spike. The occupation frequencies of this subregion are therefore a direct measure of the number of spike missed, per neuron, by the standard hybrid scheme. They are shown as red stars in Figure 9 for the various sets of network parameters (μ, σ^2^, *J*). The frequency of missed spikes does not have a simple monotonic dependence on the three parameters, owing to the interaction of several mechanisms, discussed in section 4.4.2. An increase in synaptic coupling *J* or input current μ generally leads to more frequently missed spikes because the neuron spikes more often overall. For very high—suprathreshold—input currents, however, the frequency decreases again until no spikes are missed anymore; this change in trend happens because the checkpoints become more frequent. Regimes of low *J* are diffusion-like processes, where frequent arrival of synaptic events does not lead to missed spikes. Regimes of high *J* are shot-noise processes, where sudden and infrequent arrival of synaptic events leads to suprathreshold excursions and missed spikes. Separate simulations show that the hybrid scheme based on the lossless method, with either algorithm, reproduce the analytic solution of the neuron model within floating point precision.

For biologically realistic synaptic couplings, *J* < 1 mV, the standard hybrid scheme is 30% faster than the scheme with the lossless method, and misses <1 spike every 10^6^ test calls, per neuron; for very low couplings *J* < 1 mV this figure even becomes <1 spike every 10^8^ test calls. For synaptic couplings *J* > 1 mV the standard hybrid scheme starts to miss more spikes, reaching even one missed spike every 500 test calls for subthreshold average input currents; and it is only 8% faster than the hybrid scheme with the lossless method. From these figures a user can decide to use the hybrid scheme with the standard or the lossless test, depending on the desired balance of accuracy and speed.

## 5. Summary and discussion

We have presented a general method to detect threshold crossing for an integrable, affine or linear neuronal dynamical law. This method is based on the geometric idea of propagating the threshold plane backwards in time and to determine whether the swept volume contains the initial state, rather than checking whether the membrane potential of the forward-evolved state has a maximum above threshold. These two procedures are mathematically equivalent but geometrically different. The forward-propagation procedure geometrically checks for the intersection of a curve (the state trajectory) with a hypersurface (the threshold). Our backward-propagation procedure geometrically checks for the “intersection” of a point (the state) with a hypervolume (the threshold trajectory), i.e., the inclusion of the former in the latter. This geometric view translates into a set of inequalities that an initial state has to satisfy in order not to cross the threshold during its evolution; and vice versa, if the system is not satisfied, a threshold crossing is certain. If In the latter case its time of occurrence is calculated by standard root-finding algorithms (Press et al., 2007, ch. 9).

We have calculated the system of inequalities expressing the threshold-crossing condition for a generic affine or linear neuronal dynamical law in any dimension. The result is the conjunction of inequalities (Equation 25). It consists of two linear inequalities in the state-space variables, voltage and currents, and a non-linear one. The numerical implementation of this system of inequalities can be further optimized, on a case-by-case basis. In order to give a concrete implementation example of the generic inequalities (Equation 25), to show their geometrical meaning, and to give an example of optimization, we have applied our procedure step-by-step to the two-dimensional case of a leaky integrate-and-fire neuron with exponentially decaying post-synaptic currents (Rotter and Diesmann, 1999). The generic inequalities (Equation 25) take in this case the concrete form (Equation 46).

The quantitative data in the present work are obtained by integrating these inequalities into a combined event-and-time-driven simulation framework (Morrison et al., 2007) for large-scale spiking neuronal network models as released by Bos et al. (2015). Implementation and comparison to earlier work show that:

- the system of inequalities, the non-linear one in particular, can be expressed analytically in terms of the state-space variables even when the original threshold-crossing condition involves a transcendental equation; compare Equation (46) with Equation (31);
- the computationally expensive non-linear function in the system can be conveniently approximated by a simplicial mesh, speeding up the algorithm even further by testing a linear inequality first, ruling out the majority of initial states;
- our method reproduces the analytic solution of the neuron model within floating point precision. It detects all threshold crossings, in particular those that the approximate test (Equation 1) of Hanuschkin et al. (2010) misses in some ranges of mean activity, fluctuation, and synaptic-coupling strength (Figure 9);
- at the default spike accuracy of 0.1 ms of the reference simulator, our method is 20–30% slower than the fastest available solver with spike loss (Hanuschkin et al., 2010). It therefore compares with embedded event driven methods in speed (see Hanuschkin et al., 2010, Figure 5, inset).

In practice the method of Hanuschkin et al. (2010) rarely misses spikes for biologically realistic synaptic amplitudes. At low frequencies of afferent synaptic events, however, threshold crossings can be missed, because fewer incoming events mean fewer checkpoints on the grid, and more time available for the voltage of a neuron to surpass threshold and go below it again. Our method offers an alternative for users who need exact spike detection and are willing to pay a price in terms of a slightly longer computation time.

In many simulations the time constants of excitatory and inhibitory currents are different, because these currents are mediated by various receptors, each with its characteristic time scale. The state of such a model is a vector in a three-dimensional state space, its variables being the excitatory and inhibitory currents and the membrane voltage. If the dynamical law of such model is still affine or linear, the general method we have here presented can still be applied to find the threshold crossings without losses. The threshold is in this case a two-dimensional flat surface, and its back-propagated image is also a flat surface within the three-dimensional space, as shown in Figure 4.

In state spaces of higher dimensions it can be more difficult to derive the non-linear inequality of the curved envelope in the system (Equation 25) in closed form, but it is still possible to construct a nested sequence of linear inequalities, corresponding to a flat simplicial mesh over the curved envelope, that approximate the non-linear inequality to any desired precision, as explained in section 3.2.3 and exemplified in section 4.4.1. Such construction needs to be done only once for any given model, prior to the simulation, and it cannot miss spikes owing to the concavity of the envelope. Being linear, such nested inequalities are likely computationally not too expensive, but further work must investigate the scaling of the computational cost of our method in such cases. The model considered here presents the simplest non-trivial case in which our algorithm can be applied.

At the same time, the step from a model without synaptic filtering to one with synaptic filtering is of qualitative nature: the response properties of the two models are fundamentally different, for example in terms of the weak signal transmission in the limit of high frequencies (Brunel and Wang, 2001). As such the described model is relevant, because it remedies some artifacts of its simpler counterparts.

An important extension of the method presented here is the inclusion of non-linear sub-threshold dynamics, such as in Hodgkin-Huxley dynamics, the quadratic integrate and fire model, or the exponential integrate-and-fire model (Gerstner and Kistler, 2002). Having a non-linear sub-threshold dynamics does not invalidate the general idea we have used, but makes it more complex to derive a system of inequalities separating the states that lead to spikes from those who do not. In principle the idea of back-propagation of the threshold plane can still be applied to a non-linear dynamical law, although the states on the threshold, when backpropagated, will not lie on a hyperplane. The hypersurface separating the spike and no-spike regions is still a set of critical points given by the vanishing of the Jacobian determinant of the backpropagation map, as in section 3.2.1. The uniqueness of the solution of differential equations guarantees the validity of this approach. In practice the resulting mathematical expressions will be analytically tractable only in very special cases. Yet, the resulting envelope could still be found with numerical methods—as we said this only needs to be done once per simulation—and it could be approximated by a mesh of simplices or splines.

Most models with non-linear sub-threshold evolution equations, however, do not require the approach described here in the first place. The reason is that the non-linearity in these models typically causes the generation of an explicit action potential, the rapid increase (or divergence in some models) of the membrane potential. This is certainly true for the examples mentioned above. The very threshold-crossing problem that we have solved here for the simplified leaky integrate-and-fire dynamics is therefore absent in these more biophysically realistic models at the expense of having to solve a non-linear set of differential equations at each time step.

The problem of detecting some sort of threshold crossing in a system of coupled first-order linear differential equations appears in many other applications and phenomena like switching, friction, and saturation (Hiebert and Shampine, 1980). For example, in an air-conditioning unit a thermostat controls the on-off state based on a certain threshold value of the room temperature (Shampine, 1994). The dynamics of this system is similar to that described in section 4.1. Another example is the problem of ejecting a pilot such that collision with the aircraft stabilizer is avoided.

The present work has used concepts from differential geometry, in particular extrusions (Bossavit, 2003) and critical points of maps between manifolds, and shows that these concepts have a readily understandable geometrical and visual meaning. The notion of extrusion has recently found applications in numerical and discretization techniques for partial and integral differential equations (Desbrun et al., 2005). The notion of critical points of a manifold mapping is ubiquitous in science: from the caustics of propagating seismic fronts, at which the seismic wave changes its phase (Romanowicz and Dziewonski, 2007) § 1.04, to the singularities between two coordinate charts in general relativity (Misner et al., 2003), which affect the accuracy of global navigation satellite systems (Coll et al., 2012; Sáez and Puchades, 2013).

Indeed, the neuron model analyzed in section 4 exhibits a similarity with the dynamics of a point mass near a black hole. If the simulation timestep *h* is very large the curved surface separating the states that lead to a spike from those that do not acts like an event horizon in general relativity: a state evolved from the spike region can enter the no-spike region, but once there it cannot escape and will always remain a “no-spike” state. This is only true for the dynamical law (Equation 7), though, with constant affine term and no resets at threshold. Inputs from other neurons lead to discontinuous changes in the affine term of the dynamical law, causing a “transport” of initial states out of the event horizon, from the no-spike to the spike region. Nevertheless, such similarities are maybe more than mere coincidences. For example, the trajectory of the threshold surface of a leaky integrate-and-fire model with α-shaped post-synaptic currents (Bernard et al., 1994) can be implicitly expressed in terms of the Lambert-*W* function (Corless et al., 1996), as an analysis along the lines of section 3.1 shows; and this function also appears in the implicit expression of point-mass trajectories in (1+1)-dimensional general relativity (Mann and Ohta, 1997). It is surely worthwhile to bring the younger field of neuronal dynamics closer to ideas and techniques of the elder fields of differential geometry and general relativity.

## Author contributions

All authors listed have made a substantial, direct and intellectual contribution to the work, and approved it for publication.

### Conflict of interest statement

The authors declare that the research was conducted in the absence of any commercial or financial relationships that could be construed as a potential conflict of interest. The reviewer MD declared a shared affiliation, though no other collaboration, with the authors to the handling editor, who ensured that the process nevertheless met the standards of a fair and objective review.

## A. Appendix

### A.1. Interpolation for threshold-crossing time

If we know that the neuron voltage *V* is below threshold at times *t* and *t*+*h* but above threshold somewhere in the interval ]*t, t*+*h*], then by continuity it must reach a maximum above threshold at a time *t*_max_ ∈ ]*t, t* + *h*]. This time can be obtained solving the equation $\dot{V}\left(t_{\text{max}}\right)=0$, with *V*(*t*) given by Equation (27). The solution (Hanuschkin et al., 2010) is

(A1) $$\begin{array}{l} t_{\text{max}}=-\frac{\tau \tau_{\text{s}}}{\tau -\tau_{\text{s}}}\ln\left[\frac{\tau }{\tau_{\text{s}}}-\frac{\tau -\tau_{\text{s}}}{\tau }\left(\frac{I_{\text{e}}}{I}-\frac{CV}{\tau I}\right)\right], \end{array}$$

from which the potential *V*(*t*_max_) > θ can also be easily calculated.

The time *t*_θ_ ∈ ]*t, t*_max_] at which the first threshold crossing occurs, *V*(*t*_θ_) = θ, must lie between *t* and *t*_max_, with *V*(*t*) < *V*(*t*_θ_) < *V*(*t*_max_), and can thus be interpolated using a root-finding algorithm (Press et al., 2007).

### A.2. Network setup

The dynamics of the membrane potential *V* and synaptic current *I* can be described, if the input is treated stochastically and for weak synaptic couplings, by a diffusion process with equations (Fourcaud and Brunel, 2002)

(A2) $$\begin{array}{l} \begin{array}{cc} \tau \frac{dV}{dt} & =-V\left(t\right)+\left(RI\right)\left(t\right),\\ \tau_{\text{s}}\frac{d\left(RI\right)}{dt} & =-\left(RI\right)\left(t\right)+\mu +\sigma \sqrt{\tau }\xi \left(t\right). \end{array} \end{array}$$

with ξ a zero-mean Gaussian process. The parameters μ and σ^2^ characterize the stochastic input and are the mean and variance of the total incoming synaptic current. They are related to the synaptic couplings {*J*_*i*_} and firing rates {*r*_*i*_} of the input neurons via $\mu =\tau \underset{i}{\sum }J_{i}r_{i}$ and $\sigma^{2}=\tau \underset{i}{\sum }{J_{i}}^{2}r_{i}$. If we have one excitatory and one inhibitory population of input neurons, mimicked by two Poisson generators with rates *r*_E_ and *r*_I_ coupled to the neuron with strengths *J*_E_ and *J*_I_ (where $J=\frac{\tau }{C}w$ and *w* is the synaptic weight of the current), and by an input current *I*_e_, then

(A3) $$\begin{array}{l} \begin{array}{cc} \mu & =\frac{I_{\text{e}}\tau }{C}+\tau \left(J_{I}r_{I}+J_{E}r_{E}\right)\\ \sigma^{2} & =\tau \left({J_{\text{I}}}^{2}r_{\text{I}}+{J_{\text{E}}}^{2}r_{\text{E}}\right),\\ \frac{1}{r} & =\tau_{\text{r}}+\tau \sqrt{\pi }\int_{\frac{V_{\text{r}}-\mu }{\sigma }+\frac{\left|\zeta \left(1/2\right)\right|}{\sqrt{2}}\sqrt{\frac{\tau_{\text{s}}}{\tau }}}^{\frac{\theta -\mu }{\sigma }+\frac{\left|\zeta \left(1/2\right)\right|}{\sqrt{2}}\sqrt{\frac{\tau_{\text{s}}}{\tau }}}{\text{e}}^{y^{2}}\left[1+\text{erf}\left(y\right)\right]\text{d}y, \end{array} \end{array}$$

where *r* is the output firing rate of the neuron, approximated to linear order in $\sqrt{\tau_{\text{s}}/\tau }$, τ_r_ is the refractory time, *V*_r_ the reset voltage, and ζ the zeta function (Abramowitz and Stegun, 1972).
